# Multi-Omic Profiling of a Newly Isolated Oxy-PAH Degrading Specialist from PAH-Contaminated Soil Reveals Bacterial Mechanisms to Mitigate the Risk Posed by Polar Transformation Products

**DOI:** 10.1021/acs.est.2c05485

**Published:** 2022-12-14

**Authors:** Sara N. Jiménez-Volkerink, Joaquim Vila, Maria Jordán, Cristina Minguillón, Hauke Smidt, Magdalena Grifoll

**Affiliations:** † Department of Genetics, Microbiology and Statistics, University of Barcelona, Av. Diagonal, 643, 08028 Barcelona, Spain; ‡ Department of Nutrition, Food Science and Gastronomy, University of Barcelona, Avda. Prat de la Riba, 171, 08921 Sta. Coloma de Gramanet, Barcelona, Spain; § Laboratory of Microbiology, Wageningen University & Research, Stippeneng 4, 6708 WE Wageningen, the Netherlands

**Keywords:** oxy-PAHs, bacterial degradation, Baeyer−Villiger, 9,10-anthracenedione, PAHs, transformation products

## Abstract

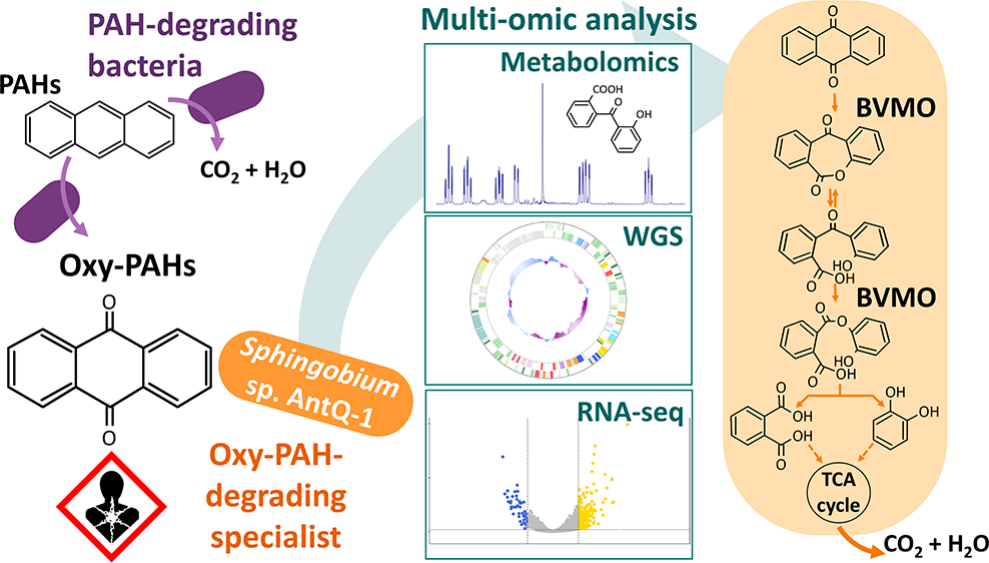

Polar biotransformation products have been identified as causative agents for the eventual increase in genotoxicity observed after the bioremediation of PAH-contaminated soils. Their further biodegradation has been described under certain biostimulation conditions; however, the underlying microorganisms and mechanisms remain to be elucidated. 9,10-Anthraquinone (ANTQ), a transformation product from anthracene (ANT), is the most commonly detected oxygenated PAH (oxy-PAH) in contaminated soils. Sand-in-liquid microcosms inoculated with creosote-contaminated soil revealed the existence of a specialized ANTQ degrading community, and *Sphingobium* sp. AntQ-1 was isolated for its ability to grow on this oxy-PAH. Combining the metabolomic, genomic, and transcriptomic analyses of strain AntQ-1, we comprehensively reconstructed the ANTQ biodegradation pathway. Novel mechanisms for polyaromatic compound degradation were revealed, involving the cleavage of the central ring catalyzed by Baeyer–Villiger monooxygenases (BVMO). Abundance of strain AntQ-1 16S rRNA and its BVMO genes in the sand-in-liquid microcosms correlated with maximum ANTQ biodegradation rates, supporting the environmental relevance of this mechanism. Our results demonstrate the existence of highly specialized microbial communities in contaminated soils responsible for processing oxy-PAHs accumulated by primary degraders. Also, they underscore the key role that BVMO may play as a detoxification mechanism to mitigate the risk posed by oxy-PAH formation during bioremediation of PAH-contaminated soils.

## Abstract 2

During the bioremediation of PAH contaminated soils, toxic transformation products may accumulate. This study demonstrates the existence of bacteria specialized in their degradation and identifies the metabolic processes involved.

## Introduction

Industrial soils related to the production, transport, storage, or use of petroleum- or coal-derived products are often impacted by contamination with polycyclic aromatic hydrocarbons (PAHs), posing a risk for human health and the environment. PAHs are embedded in complex mixtures that include many other polyaromatic components, including heterocyclic aromatic compounds and oxygenated-PAHs (oxy-PAHs).^1^ Oxy-PAHs, such as aromatic ketones, quinones, or lactones, are found along PAHs in the contaminant source but can also be readily formed due to photo-/chemical oxidation^2^ or microbial transformation of PAHs.^3,4^ Despite this, measures of risk and remediation effectiveness in PAH-contaminated sites are still based exclusively on concentration levels for the 16 regulated PAHs listed by the US-EPA in 1979, neglecting the co-occurrence and fate of other toxicologically relevant compounds.^5,6^

Bioremediation is the most sustainable technology for the clean-up of hydrocarbon contaminated sites due to its cost-effectiveness, low environmental footprint, and capability to restore key natural soil functions.^7^ Nevertheless, some studies have reported an eventual increase in genotoxicity in bioremediated PAH-polluted soils that has been associated to polar fractions enriched in oxy-PAHs resulting from PAH biotransformations.^8,9^ A recent study has identified 2*H*-naphtho[2,1,8-*def*]chromen-2-one, a bacterial metabolite of pyrene, as a main contributor of the genotoxicity observed in PAH-polluted soil after treatment in an aerobic bioreactor.^9^ Due to their physicochemical properties, oxy-PAHs have greater bioavailability and environmental mobility than PAHs,^10,11^ and a number of them have been demonstrated to present higher (geno)toxic, mutagenic, and carcinogenic activities than their unsubstituted counterparts.^12,13^ Several bacterial isolates are known to produce oxy-PAHs as dead-end products during aerobic metabolism of PAHs.^14,15^ Further biodegradation of such oxy-PAHs has been reported after stimulation of the microbial activity in soils;^3,4^ however, little is known about the microorganisms and mechanisms underlying their fate in the environment.

9,10-Anthraquinone (ANTQ), the ready oxidation product from anthracene (ANT), is one of the most commonly found oxy-PAHs in PAH-contaminated soils^1,10^ and has been classified as possibly carcinogenic to humans (group 2B) by the International Agency for Research on Cancer.^16^ ANTQ has been reported as a dead-end transformation product of anthracene by pyrene-degrading mycobacteria,^14,17^ and its formation and eventual removal during biological treatment of contaminated soils has been reported.^3,18,19^ In a PAH-contaminated soil from a former manufactured-gas plant, ANTQ biodegradation was associated to uncultured *Sphingomonas* and *Phenylobacterium* species^20^ detected by DNA stable-isotope probing, but the specific metabolic mechanisms involved remain to be elucidated.

In this study, we report the isolation of a bacterial strain (AntQ-1) able to utilize 9,10-anthraquinone as a sole carbon source from PAH-contaminated soil from a historical wood-treating facility in the south of Spain. This isolate is used as a model to shed light on the microbial mechanisms driving oxy-PAH biodegradation in contaminated soils. Combining metabolomic, genomic, and transcriptomic analyses, we have comprehensively elucidated the metabolic pathway and identified the key genes involved in the biodegradation of anthraquinone. The environmental relevance of bacterial strain AntQ-1 and its degradative mechanisms are confirmed by monitoring the identified genes during microcosm incubations of PAH-contaminated soil.

## Materials and Methods

### Chemicals, Media, and Cultivation Conditions

Anthracene (ANT, 99% purity), 9,10-anthraquinone (ANTQ, 97%), 9-anthracenone (ANTO, 97%), and all other aromatic compounds were purchased from Sigma-Aldrich Chemie (Germany). All solvents were obtained from J.T. Baker (The Netherlands) with the highest purity available (organic residue analysis grade). Diazomethane was generated by alkaline decomposition of Diazald (*N*-methyl-*N*-nitroso-*p*-toluenesulfonamide). Strain AntQ-1 was grown either in mineral medium (MM)^21^ or Reasoner’s 2A (R2A) supplemented with vitamin B_12_ (50 μg L^–1^) (B_12_-MM or B_12_-R2A). ANTQ (0.1 g L^–1^) was supplied in acetone solution to solid culture media before plating. In liquid media, ANTQ and other aromatic substrates were added to sterile empty flasks in dichloromethane solution, and the solvent was completely evaporated before adding the sterile liquid medium. Microcosms and liquid cultures were incubated at 25 °C under rotary shaking (150 rpm).

### Biodegradation of ANT and ANTQ in Sand-in-Liquid Soil Microcosms

Sand-in-liquid microcosms^22^ consisted of 20 mL glass vials prepared with 6 mL of MM and 3 g of sand (Panreac, Spain) coated with 0.1 g L^–1^ of either ANT or ANTQ. Inoculated microcosms received 4 mL of MM and 2 mL of a preincubated soil suspension as inoculum. Uninoculated controls were prepared with 6 mL of sterile mineral medium. Series of triplicate microcosms for each substrate were incubated during 0, 5, 10, 15, 20, 25, and 30 days (Table S1). At each incubation time, a set of triplicates was solvent-extracted to quantify residual ANT or ANTQ, as described below. Another set of triplicates was used to obtain 1 mL of sand and liquid samples for total DNA and RNA extraction (Supplementary Material). 16S rRNA PCR amplification, denaturing gradient gel electrophoresis (DGGE) analysis, excision of selected bands, and sequencing were performed as described elsewhere.^22^

The creosote-polluted soil, collected from a historical wood-treating facility in southern Spain, had a high concentration of polycyclic aromatic compounds (PACs). In total, 17 PAHs (16 US EPA PAHs + benzo(*e*)pyrene), 7 oxy-PAHs, and 7 N-PACs were quantified: Σ17 PAHs = 25,791 ± 2112 ppm, Σ7 oxy-PAHs = 273 ± 9 ppm, and Σ7 N-PACs = 2568 ± 366 ppm (Table S2). Before inoculation of the sand-in-liquid microcosms, the soil was pre-incubated to reduce the concentration of native PAHs. Fifty grams of the soil were combined with 100 g of sand and 200 mL of MM, and the mixture was incubated for 21 days, resulting in a 93% reduction in the concentration of native Σ17 PAHs, 52% of Σ7 oxy-PAHs, and 85% of Σ7 N-PACs. A 1/10 dilution of this preincubated soil suspension was used to inoculate the microcosms, being the final concentration of all native PACs below 6 ppm.

For chemical analysis, the liquid and sand phases of the microcosms were separated by filtration (Whatman No. 1). The aqueous phase was extracted with 3 × 10 mL dichloromethane, while the solid phase was combined with 3 g of Na_2_SO_4_ and extracted in an ultrasonic bath first with 2 × 10 mL dichloromethane:acetone (2:1) and then with 10 mL dichloromethane. Extracts from the aqueous and solid phases were combined and concentrated prior to analysis by GC. For details on chemical analysis, see the Supplementary Material. Degradation rates were calculated by dividing the difference in molar concentration between two time points by the difference in time between those time points in days.

### Isolation of the 9,10-Anthraquinone-Degrading Strain *Sphingobium* sp. AntQ-1

Strain AntQ-1 was isolated from the ANTQ-spiked sand-in-liquid microcosms. Serial dilutions from 30 day microcosms were inoculated on solid MM containing yeast extract (YE, 0.25 g L^–1^) and 9,10-anthraquinone (0.1 g L^–1^). Plates were prepared with and without vitamin B_12_ (50 μg L^–1^) since B_12_ auxotrophy is common in soil bacteria. After 30 days of incubation, colonies surrounded by clearing areas, indicative of ANTQ degradation, were only detected on B_12_-MM agar plates and were then selected and purified on B_12_-R2A agar plates. Growth on easily assimilable carbon sources (0.5 g L^–1^), including acetate, lactate, pyruvate, glucose, arginine, and glutamine, was tested in triplicate 5 mL liquid MM cultures with and without vitamin B_12_ by measuring optical density (600 nm).

Strain AntQ-1 was identified on the basis of its 16S rRNA gene sequence. Genomic DNA was purified using InstaGene Matrix (Bio-Rad, CA, USA), and the nearly complete 16S rRNA gene sequence (approximately 1350 bp) was obtained by sequencing the fragment amplified with primers 27F and 1492R.^23^ The 16S rRNA gene sequence is available in GenBank under accession number ON097133. PCR amplification, purification, and sequencing procedures are described in the Supplementary Material. Strain AntQ-1 has been deposited in the Spanish Type Culture Collection under accession code CECT 30664.

### Utilization of PAHs, Oxy-PAHs, and Aromatic Carboxylic Acids by *Sphingobium* sp. AntQ-1

ANTQ utilization as sole carbon and energy source was tested in 20-mL B_12_-MM liquid cultures with ANTQ (0.1 g L^–1^) as sole carbon source. Uninoculated flasks and flasks without ANTQ served as controls. At given incubation times for a period of 10 days, the entire content of the triplicate flasks was used to determine the protein concentration using a modified Lowry method.^24^ Another set of triplicates was extracted with dichloromethane (5 × 10 mL), and their ANTQ concentration was analyzed by GC-FID.

Growth on other aromatic compounds (0.1 g L^–1^) was demonstrated by an increase in cell-protein in triplicate 5 mL B_12_-MM liquid cultures with respect to uninoculated controls incubated for 10 days. Tested compounds included fluorene, anthracene, phenanthrene, benz(*a*)anthracene, 9-fluorenone, anthrone, 9,10-phenanthrenequinone, 7,12-benz(*a*)anthracenequinone, 5,12-naphthacenequinone, xanthone, indanone, carbazole, acridine, dibenzofuran, dibenzosuberone, benzophenone, 2-methylanthraquinone, bisphenol A, ethinylestradiol, anthraquinone-2-carboxylic acid, benzoic acid, diphenic acid, phthalic acid, 1-hydroxy-2-naphthoic acid, cinnamic acid, protocatechuic acid, carboxybenzaldehyde, benzenetricarboxylic acid, toluic acid, hydroxycoumarin, catechol, salicylic acid, and 1,8-naphthalic anhydride. Growth on naphthalene, camphor, and biphenyl was tested in B_12_-MM plates with those substrates as crystals on the lid of the plates.

All cultures were inoculated (1%) with a suspension of strain AntQ-1 cells pregrown in 5 mL of B_12_-R2A medium during 48 h (*A*_600 =_ 0.9) and washed thrice with MM.

### Identification of 9,10-Anthraquinone Metabolites

Metabolites were identified in washed-cell suspensions incubated with ANTQ crystals. Strain AntQ-1 was pregrown in 4× 2-L Erlenmeyer flasks containing 400 mL of B_12_-MM with YE (0.25 g L^–1^) and ANTQ crystals (0.5 g L^–1^). At the late exponential phase (72 h), remaining ANTQ crystals were removed by filtration through sterile glass wool and cells were harvested by centrifugation, washed twice and resuspended in 400 mL of phosphate buffer (50 mM, pH 7). Cell suspensions were incubated with ANTQ crystals (0.5 g L^–1^) in a 2 L Erlenmeyer flask. Controls without cells were included to assess abiotic degradation. After 48 h, when maximum metabolite accumulation was observed, according to previous experiments, cells and residual ANTQ crystals were removed by centrifugation and supernatants were extracted with 5 × 100 mL ethyl acetate and then acidified to pH 2.5 (6 N HCl) and extracted again in the same manner. Metabolites in neutral and acidic extracts were identified by gas chromatography coupled to mass spectrometry (GC–MS) and by high pressure liquid chromatography coupled to high-resolution mass spectrometry with electrospray ionization (HPLC–ESI–HRMS). Structural elucidation of metabolite II was achieved by nuclear magnetic resonance (NMR). Metabolites from 9-anthrone were identified using the same experimental conditions. Technical details and conditions for the chemical analyses are provided in the Supplementary Material.

### De Novo Whole Genome Sequencing

Genomic DNA of strain AntQ-1 was extracted with the MasterPure Gram Positive DNA Purification kit (Lucigen, USA), with O/N lysis, from a 1 mL aliquot of a culture grown in B_12_-R2A medium for 48 h. DNA quality was checked by agarose gel electrophoresis, and DNA concentration was measured by Qubit (Thermo Fisher, USA). Whole genome sequencing was performed at Novogene Co. (Beijing, China) on both an Illumina HiSeq (Illumina, CA, USA) platform, to generate 150 bp paired-end reads, and a PacBio Sequel (Pacific Biosciences, CA, USA) platform. Illumina paired-end reads were analyzed with FastQC (v0.11.8), and Trimmomatic (v0.38) was used for adapter removal and quality filtering. PacBio raw reads were filtered and processed using SMRT Link (v.9.0). Hybrid de novo assembly using Illumina and PacBio subreads was performed with Unicycler (v0.4.8), with default parameters. The quality assessment of the genome assembly was done using QUAST (v.5.0.2), and genome assembly graphs were visualized with Bandage (v.0.8.1). The genome sequence of strain AntQ-1 was annotated with Prokka (v.1.14.5). The predicted proteins were assigned to orthologous groups and mapped to KEGG pathways using the KEGG Automatic Annotation Server (KAAS). Completeness of phthalate and catechol metabolic pathways was confirmed using MetaCyc (https://metacyc.org/). The presence of genes related with PAH metabolism (aromatic ring mono- and dioxygenases) was assessed using Prokka, RAST, and KEGG annotation and searches against the AromaDeg database (http://aromadeg.siona.helmholtz-hzi.de). The genome assembly is available at the NCBI Genome repository under BioProject ID PRJNA821003 (BioSample SAMN27034154) with accession numbers CP094976 to CP094983.

### RNA Sequencing

Cells of *Sphingobium* sp. AntQ-1 grown in B_12_-R2A medium were washed twice with MM and used to inoculate triplicate 100 mL cultures of medium B_12_-MM with either ANTQ or acetate (0.1 g L^–1^) as the sole carbon source. At the mid-exponential phase (54 h), the cells were harvested by centrifugation and snap-frozen in liquid nitrogen for subsequent RNA extraction. Total RNA was isolated by a method combining the TRIzol reagent (Invitrogen, CA, USA) and the RNeasy Mini kit (QIAGEN, Germany). In brief, 4 μL of β-mercaptoethanol and 0.4 mL of buffer RLT were added to 1 mL of TRIzol containing the bacterial pellet. The mixture was transferred to a tube containing 0.8 g of glass beads (diameter 0.1 mm), followed by three times of bead beating for 1 min at 5.5 m s^–1^, with ice cooling steps in between. Subsequently, 0.2 mL of ice-cold chloroform was added. The solution was mixed gently followed by centrifugation at 12,000*g* for 15 min at 4 °C. RNA isolation was continued with the RNA clean-up using the RNeasy Mini kit according to the manufacturer’s instructions. Genomic DNA was removed by an on-column DNase digestion step during RNA purification (DNase I recombinant, RNase-free, Roche Diagnostics, Germany). RNA concentration was measured by Qubit (Thermo Fisher, USA), and RNA quality was assessed by a Qsep Bioanalyzer (BiOptic Inc., Taiwan).

Ribosomal RNA removal, library preparation, and Illumina NovaSeq 6000 2 × 150 bp sequencing was performed by Novogene Co. (Beijing, China). Raw read data quality was inspected using FastQC (v0.11.8) and trimmed with Trimmomatic (v0.38). Trimmed reads were mapped against the genome sequence of *Sphingobium* sp. AntQ-1 using bwa-mem (v0.7.17), and the quality of the mapping was analyzed with the flagstat command of samtools (v1.9). Mapped reads were quantified with the featureCounts function of the Subread package (v2.0.1). Normalization of counts and differential gene expression analysis was conducted with DESeq2.^25^ All software applications were used with default settings. Transcript abundance was defined as transcripts per million (TPM), normalized by gene length and sequencing depth. Data from the RNA-Seq experiment has been deposited in the NCBI Gene Expression Omnibus under GEO series accession number GSE199781 (BioProject PRJNA821003), and the sample accession numbers are GSM5984243–GSM5984248.

### Quantitative PCR Analysis

Validation of the RNA-Seq analysis and quantification of relevant genes in the sand-in-liquid samples was achieved by quantitative PCR (qPCR) analysis. RNA samples from the RNA-Seq experiment were reverse-transcribed to cDNA with the High-Capacity cDNA Reverse Transcription kit (Thermo Fisher Scientific). qPCR reactions (20 μL) were carried out on an Applied Biosystems StepOnePlus Real-Time PCR system using PowerUp Sybr Green Master Mix (Applied Biosystems, USA) with 1 μL of template and 4 pmol of each primer. Primer sets were designed to target the 16S rRNA gene of *Sphingobium* sp. AntQ-1 and overexpressed genes in the RNA-Seq experiment (Table S3). All primers were designed using Primer-BLAST and experimentally validated by PCR. Relative gene expression levels were calculated using the 2^–ΔΔCT^ method.^26^ For quantification, 6-point 10-fold standard plasmid dilution series were used. Standard plasmids were obtained by cloning PCR amplification products with the pGEM-T Easy Vector System (Promega, WI, USA). Plasmids were purified with the GeneJET Plasmid Miniprep Kit (Thermo Scientific, USA), quantified using Qubit, and validated by sequencing.

## Results and Discussion

### Anthracene and 9,10-Anthraquinone Biodegradation and Microbial Community Shifts in Sand-in-Liquid Soil Microcosms Inoculated with a PAH-Contaminated Soil

Small-scale microcosms with ANT presented 70% of substrate removal in 25 days (see below in Figure 3). Degradation started after a 5-day lag phase and followed a first-order kinetics between 10 and 25 days (17.9 ± 4.9 μmol L^–1^ day^–1^). ANTQ degradation did not present a lag phase and proceeded until complete removal at day 25, reaching maximum rates during the first 10 days (27.0 ± 1.4 μmol L^–1^ day^–1^). DGGE analysis of bacterial 16S rRNA genes and transcripts showed community shifts in response to ANT and ANTQ (Figure S1). The DGGE profiles from the ANT-microcosms presented a substantial increase in the intensity of discrete bands, the most intense (B1) corresponding to a member of *Sphingobium* (99% probability). The ANTQ-microcosms presented DGGE profiles remarkably different from those of the inoculum or the ANT-microcosms, showing a new predominant band (B2) in both total and active populations at all timepoints. Sequence analysis affiliated band B2 to genus *Sphingobium* (100% probability), with only 97% similarity to band B1. These results pointed toward the existence of populations specialized in the removal of the quinone. This is in agreement with the findings from Rodgers-Vieira et al.,^20^ who, using DNA-based stable isotope probing (SIP), identified different bacteria involved in the assimilation of anthraquinone or anthracene in a PAH-contaminated soil. These authors associated *Sphingomonas* and *Phenylobacterium* species with anthraquinone degradation, while members of *Immundisolibacter* and *Sphingomonadales* were identified as the main anthracene degraders.

### *Sphingobium* sp. AntQ-1, a 9,10-Anthraquinone-Degrading Bacterial Strain

*Sphingobium* sp. AntQ-1 was isolated from the ANTQ sand-in-liquid microcosms in B_12_-MM agar plates supplemented with YE and ANTQ as the main carbon source. After 10 days of incubation, the strain produced colonies surrounded by clearing zones indicative of anthraquinone degradation (Figure S2). Phylogenetic analysis based on the 16S rRNA gene sequence using RDP Seqmatch indicated that strain AntQ-1 was most closely related to *Sphingobium rhizovicinum* type strain CC-FH12-1 (98.7% identity, NR_044226.1). BLAST search on the GenBank database identified type strain *Sphingobium cupriresistens* CU4 (99.2%, NR_109535.1) as the closest relative. The 16S rRNA gene sequence from strain AntQ-1 was identical to that of band B2 from the DGGE analysis of the ANTQ sand-in-liquid soil microcosms. Scanning electron microscopic observations of ANTQ-grown *Sphingobium* sp. AntQ-1 cells (Figure S2) revealed rod-shaped cells about 1.5 μm long and 0.4 to 0.7 μm wide, eventually aggregating around ANTQ crystals.

Utilization of 9,10-anthraquinone as a sole carbon and energy source by strain AntQ-1 was demonstrated by its removal from liquid B_12_-MM cultures along with a concomitant increase in cell protein (Figure S3). Maximum ANTQ degradation rates were observed during the first 2 days of incubation (101.4 ± 10.6 μmol L^–1^ day^–1^), with a bacterial doubling time estimated in 26 h. ANTQ was almost completely removed after 6 days of incubation (97%) when maximum protein concentration was achieved (53 ± 8.1 μg mL^–1^). To the best of our knowledge, this is the first reported bacterial isolate with the ability to grow on 9,10-anthraquinone as the sole source of carbon and energy.

A variety of other polyaromatic compounds and known PAH metabolites were tested as growth substrates (0.1 g L^–1^) in B_12_-MM cultures. Significant growth was only observed with anthrone (33.8 ± 1.7 μg protein mL^–1^), 2-methylanthraquinone (56.8 ± 1.9 μg mL^–1^), catechol (12.8 ± 5.3 μg mL^–1^), protocatechuic acid (51.8 ± 6.4 μg mL^–1^), and benzoic acid (40.2 ± 5.6 μg mL^–1^). The strain also grew on B_12_-MM with acetate, lactate, pyruvate, or glucose. Growth was never observed in MM in the absence of vitamin B_12_, confirming an auxotrophy for this vitamin. It is worth noting that strain AntQ-1 was not able to grow on ANT, the parent PAH of anthraquinone and anthrone. This specialization in oxy-PAH degradation capability had also been observed for a 9-fluorenone-degrading strain of *Pseudomonas mendocina* MC2, isolated from PAH-contaminated river and unable to grow on fluorene or any other PAH.^27^ This again would suggest the existence of populations highly specialized in oxy-PAH utilization in PAH-contaminated soils and sediments.

### Detection and Identification of 9,10-Anthraquinone Metabolites

The HPLC analysis of both the neutral (2 mg, dry residue) and acidic (3 mg) extracts from washed-cell incubation fluids of strain AntQ-1 with ANTQ revealed a major product (II, Rt 16.5 min) with a UV–visible spectrum showing λ_max_ at 210, 258, and 330 nm. Its HPLC-ESI(+)-HRMS spectrum produced the molecular formula C_14_H_11_O_4_ ([M+H]^+^ = 243.0649), with ion fragments at *m/z* 243.0649 (M^+^+H^+^, 70%), 225.0545 (M^+^-H_2_O+H^+^, 100%), and 149.0236 (M^+^-H_2_O-C_6_H_4_+H^+^, 25%) (Table S4). Metabolite II was detected as its methyl ester derivative in the GC–MS analysis of the diazomethane-treated neutral (42.9%, relative abundance) and acidic (89%) extracts. Based on the exact mass and fragmentation pattern, metabolite II was tentatively identified as 2-(2-hydroxybenzoyl)-benzoic acid. The underivatized neutral extract, however, presented a major product (I) with an MS spectrum consistent with a lactone resulting from the dehydration of metabolite II, dibenz[*b,e*]oxepin-6,11-dione (21.9%). These results suggest either that a portion of metabolite II was extracted in neutral conditions and recircularized in the GC or that products I and II were both in the culture.

Identification of metabolite II as 2-(2-hydroxybenzoyl)-benzoic acid was confirmed by NMR analysis of the acidic extract. The monodimensional ^1^H NMR spectrum was in perfect agreement with the suggested structure. The coupling pattern shown for all signals permitted the univocal assignment of signals in this spectrum to the diverse proton atoms in the structure (Figure S4 and Table S5). Bidimensional gCOSY and gHSQC spectra were also obtained (Figures S5 and S6). The gCOSY experiment confirmed the assignment of the monodimensional ^1^H NMR spectra, while the gHSQC identified the chemical shifts corresponding to protonated carbon atoms in the molecule. These values were in good agreement with the suggested structure.

The identified 2-(2-hydroxybenzoyl)-benzoic acid has been previously reported in the biodegradation of anthracene via anthraquinone by ligninolytic fungi and was attributed to the action of manganese peroxidases.^28,29^ In bacteria, the cleavage of the central ring in aromatic quinones has been observed for anthraquinone analogues and other oxy-PAHs. The *Sphingomonas xenophaga* strain QYY converted 1-aminoanthraquinone-2-sulfonic acid, a substituted anthraquinone used as a dye intermediate, to two isomers of the corresponding ring-fission acid and phthalic acid.^30^ Similar reactions were proposed for the transformation of benz(*a*)anthracene via 7,12-benz(*a*)anthracenequinone by *Mycobacterium vanbaalenii* PYR-1, with further oxidation of the quinone leading to cleavage of the central ring.^15^

In addition to metabolite II, the acidic extract presented a less abundant metabolite (III) identified as phthalic acid based on its spectral characteristics and by comparison with authentic material. This product was detected as its dimethyl ester derivative in the GC–MS analysis of the acidic extract treated with diazomethane (11%), whereas in the HPLC–ESI(+)–HRMS analysis, the detected accurate mass ([M + H]^+^ = 149.0234) corresponded to phthalic anhydride. Phthalic acid was the only metabolite detected during the growth of strain AntQ-1 on anthraquinone, but its accumulation was only transient (data not shown).

When incubated with anthrone, the ketone derivative of anthracene, *Sphingobium* sp. AntQ-1 produced anthraquinone and metabolites II and III (Table S6). This indicates that the strain is able to perform a monooxygenasic attack on the methylenic carbon of the central ring of anthrone to produce anthraquinone, which is then metabolized by the reactions described above. The fact that the strain is not able to perform a similar oxidation on the unsubstituted parent PAH may be due to the aromaticity of the central ring of ANT.

### Genomic and Transcriptomic Analyses of *Sphingobium* sp. AntQ-1

The complete genome sequence of strain AntQ-1 was obtained combining Illumina and PacBio sequencing technologies. Illumina sequencing yielded 7,612,734 paired-end reads of 150 bp with a total average Phred quality score of 35, resulting in a sequencing coverage of over 200×. For PacBio, a total of 183,593 reads were generated with an average read length of 8,544 bp and N50 9,944 bp. The final assembly had a total length of 4,904,521 bp with an average G + C content of 63.6%, consisting of eight circular contigs that corresponded to two chromosomes and six plasmids: *chr1* (3,758,784 bp, 64.0% G + C), *chr2* (720,548 bp, 63.0% G + C), *pANTQ-1* (201,917 bp, 60.6% G + C), *pl2* (80,951 bp, 61.6% G + C), *pl3* (53,641 bp, 60.6% G + C), *pl4* (51,841 bp, 63.5% G + C), *pl5* (20,675 bp, 58.9% G + C), and *pl6* (16,164 bp, 57.3% G + C) (Figure S7). Whole genome sequence comparisons confirmed the identity of strain AntQ-1 (Figure S8), showing the highest ANI score (96.49%) with *S. cupriresistens* CU4^T^ (GCA_004152865.1). The annotated genome contained 4741 coding DNA sequences (CDSs), 63 tRNA genes, and 12 rRNA genes. Genes for PAH ring-hydroxylating dioxygenases were not detected, which is consistent with strain AntQ-1 not being able to grow on this type of compounds. The genome contained six genes annotated as ring hydroxylating dioxygenases for monoaromatic compounds such as catechol, phthalic acid, benzoic acid, or benzene.

To elucidate the key functional genes associated to the ANTQ biodegradation pathway, we performed a comparative transcriptomic analysis by RNA-Seq in cultures of strain AntQ-1 with ANTQ versus cultures in acetate as control condition (Figure S9). For each growth condition, we obtained three replicates of rRNA-depleted RNA preparations. The number of clean paired-end reads averaged at 7.6 and 9.8 million, and the percentage of reads mapping to the genome was over 98.6% and 98.8% for all replicates in ANTQ and acetate conditions, respectively. The percentage of reads assigned to genomic features was over 83.8% for ANTQ RNA samples and over 88.7% for acetate RNA. Differential expression analysis yielded 180 upregulated genes in the presence of ANTQ and 45 genes with acetate, considering log2 fold change of ≥1.5 or ≤−1.5 and *p*-value of ≤0.01 (Figure S10). Upregulated genes presented a fold change of between 1.5 and 5.9, and downregulated genes presented a fold change of between −1.5 and −5.1.

**Figure 1:**
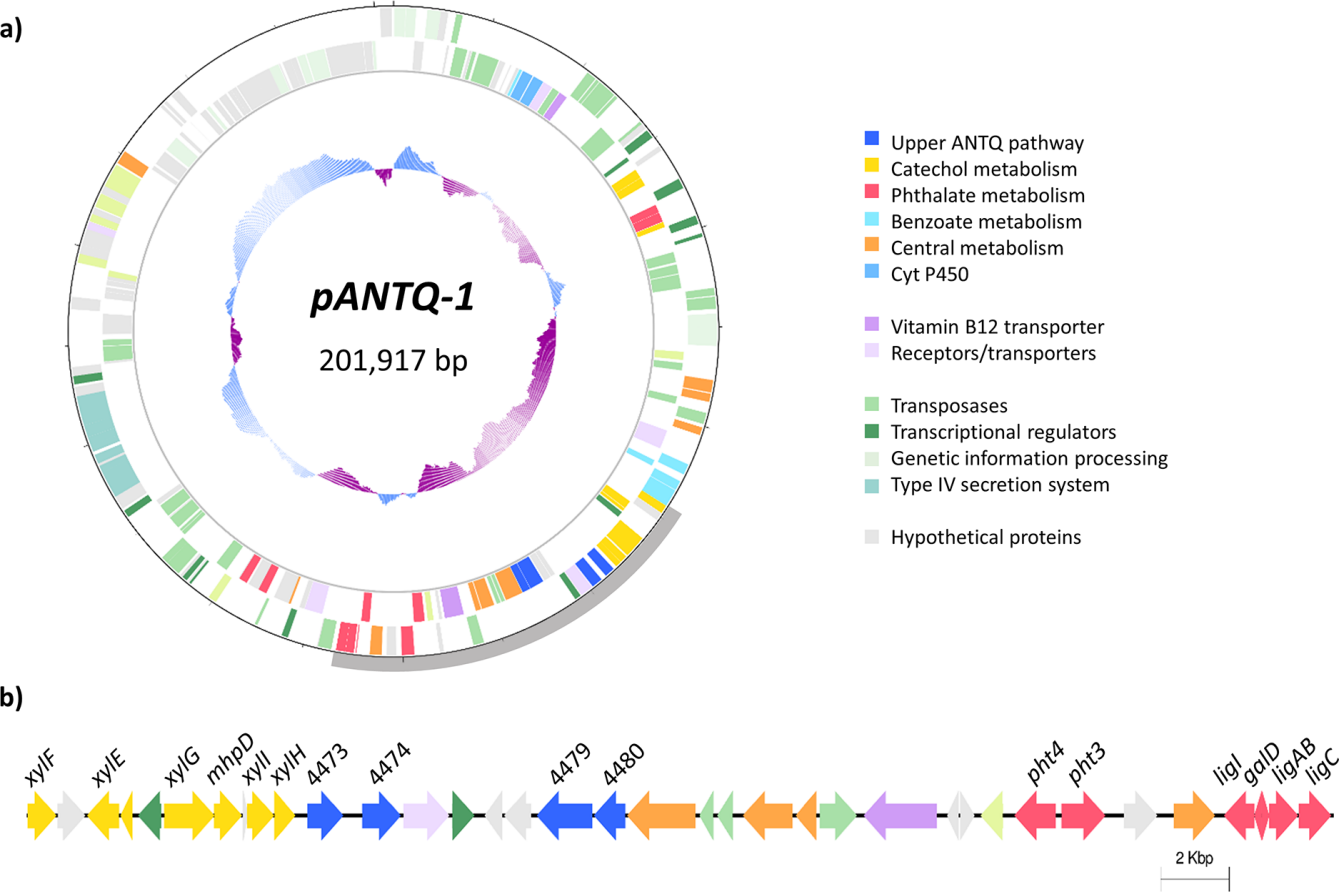
(a) Structure of plasmid *pANTQ-1* in *Sphingobium* sp. AntQ-1, encoding most of the genes related to the 9,10-anthraquinone biodegradation pathway. Going inward, the first two circles denote genes in the forward and reverse strands colored based on gene function classification. The inner circle represents GC content (above average in blue and below average in purple). Dashes in the outermost circle are placed every 10 kbp. (b) Arrangement of genes related to 9,10-anthraquinone biodegradation in plasmid *pANTQ-1* of *Sphingobium* sp. strain AntQ-1 between the positions 68,745 and 106,725 bp highlighted in gray in the plasmid map.

Among the most highly upregulated genes during growth on ANTQ (Table S7), a gene encoding a luciferase-like flavin-dependent monooxygenase (sphantq_4473) presented the highest transcript per kilobase million (TPM, 21,461) count with a fold-change of 4.05. Contiguous to it, a gene coding for a hydrolase-like protein (sphantq_4474) presented a 3.49-fold change and 2,570 TPM. Both genes were located in the megaplasmid *pANTQ-1* (Figure 1). The significant overexpression of this cluster of genes in the presence of ANTQ suggests their role in the biodegradation of this oxy-PAH. Based on the identified metabolites and the annotation of these genes, it seems that the attack on ANTQ would be initiated by a Baeyer–Villiger (BV) oxidation and subsequent hydrolysis of the lactone. Thus, gene sphantq_4473 would probably encode a Baeyer–Villiger monooxygenase (BVMO) as these are flavoenzymes that catalyze the BV oxidation of ketones and cyclic ketones to esters or lactones by inserting one atom of molecular oxygen into a C–C bond adjacent to the carbonyl group using NAD(*P*)H as the reducing agent.^31^ The presence of a bacterial luciferase-like protein domain indicates that it would probably be classified as a type II BVMO, which consists of two distinct polypeptides, the oxygenating component that binds FMN as cofactor, and the reducing component that uses NADH as cofactor. To date, the only reported type II BVMOs are two diketocamphane monooxygenases from *Pseudomonas putida* ATCC17453 involved in camphor metabolism.^32^ Phylogenetic analysis of representative type I and type II BVMOs (Figure S11) clustered sphantq_4473 together with those two known type II BVMOs, thus supporting that it belongs to this group. Blastp analysis revealed that the amino acid sequence of sphantq_4473 was closely related to flavin-dependent oxidoreductases belonging to the genus *Ramlibacter* (39–42% identity; Figure S11). The hydrolase-encoding gene sphantq_4474 was initially annotated as a hypothetical protein; however, InterProScan analysis showed the presence of an alpha/beta hydrolase_6 protein domain, and its amino acid sequence is closely related to an alpha/beta hydrolase of *Bradyrhizobium* sp. WSM3938 (61% identity).

Within plasmid *pANTQ-1*, located only 3 kb away from this gene cluster but on the reverse strand, another cluster composed by a gene coding for a BVMO (sphantq_4479) and a gene encoding a monoterpene ε-lactone hydrolase (sphantq_4480) revealed significant upregulation. Although the expression level was considerably lower than that of the first BVMO–hydrolase cluster, upregulation in ANTQ cultures could indicate a relevant role in the biodegradation of this compound. This BVMO displayed type I BVMO hallmarks: two Rossman motifs (GxGxx[G/A]) flanking two BVMO fingerprints ([A/G]GxWxxxx[F/Y]P[G/M]xxxD and FxGxxxHxxxW[P/D]) and shared sequence homology with NAD(P)/FAD-dependent oxidoreductases of diverse sphingomonads (66–67% identity; Figure S11). Type I BVMO is the most extensively studied class of BVMOs for their potential use as biocatalysts in the biosynthesis of secondary metabolites with antibiotic or anti-cancer activity as well as for their ability to catalyze key reactions in metabolic pathways of a wide range of carbon sources such as linear alkanones or alkanals and terpenoids or alicyclic and aromatic ketones.^33^

As expected from the identification of phthalate as a major anthraquinone metabolite, genes encoding for the complete phthalic acid metabolic pathway were overexpressed in ANTQ cultures. Significantly higher expression levels were observed for the *pht2345* genes, involved in the initial steps leading to the formation of protocatechuate, with fold changes ranging from 1.59 to 2.36. Most genes catalyzing the lower pathway (*ligABCI* and *galD*) were duplicated in the strain AntQ-1 genome, one copy being located in the secondary chromosome *chr2* and the other in the megaplasmid *pANTQ-1*. Overexpression was mainly detected for the genes located in the secondary chromosome, which could be attributed to the lack of *ligJK* genes in the plasmid.

Interestingly, upregulation of genes coding for both the complete catechol *meta*- and *ortho*-cleavage pathways was remarkably high in the presence of anthraquinone. The *meta*-cleavage pathway, catalyzed by *xylEFGHI* and *mhpDEF* genes encoded on plasmid *pANTQ-1*, presented fold-change levels between 1.38 and 4.57 along with significant TPM counts. Genes encoding for the *ortho*-pathway, including *catABCD, pcaDI*, *and fadA*, were found on the primary chromosome *chr1* and showed lower expression levels, although fold-change of the *catABCD* genes was relatively high (2.1–2.4). This is the second report for the catabolism of catechol by both extra- and intradiol ring cleavage in sphingomonads as Maeda and colleagues^34^ provided the first evidence for intradiol cleavage of catechol by chemical and genomic analysis during biodegradation of low molecular weight PAHs in *Sphingobium barthaii* KK22.

It is worth mentioning that a gene encoding for a BtuB vitamin B_12_ transporter (sphantq_4487) was significantly upregulated in the presence of ANTQ, with a 1.87-fold change and 3,413 TPM. This gene is located on plasmid *pANTQ-1* adjacent to the two BVMO–hydrolase clusters. Reconstruction of the biosynthetic pathway of vitamin B_12_ in the genome of strain AntQ-1 revealed its incompleteness (Figure S12) as genes for the biosynthesis of the corrin ring were missing (*cobIZGJMFKLH*), corroborating the auxotrophy of strain AntQ-1 and the need for external uptake.^35^ Overexpression of the gene encoding for the B_12_ transporter together with the inability of the strain to grow in the absence of cyanocobalamin demonstrate that it is an essential co-factor for strain AntQ-1.

Accuracy of RNA-Seq data was confirmed by RT-qPCR quantification of the transcription levels of selected upregulated genes involved in the biodegradation of anthraquinone: sphantq_4473, sphantq_4474, sphantq_4479, sphantq_4480, *pht3* (sphantq_4492), *xylE* (sphantq_4465), and *catA* (sphantq_3328). Gene expression fold-change between RNA-Seq and RT-qPCR demonstrated a good correlation with almost identical trends for each selected gene (Figure S13).

### Multi-Omic Reconstruction of the 9,10-Anthraquinone Degradation Pathway

**Figure 2:**
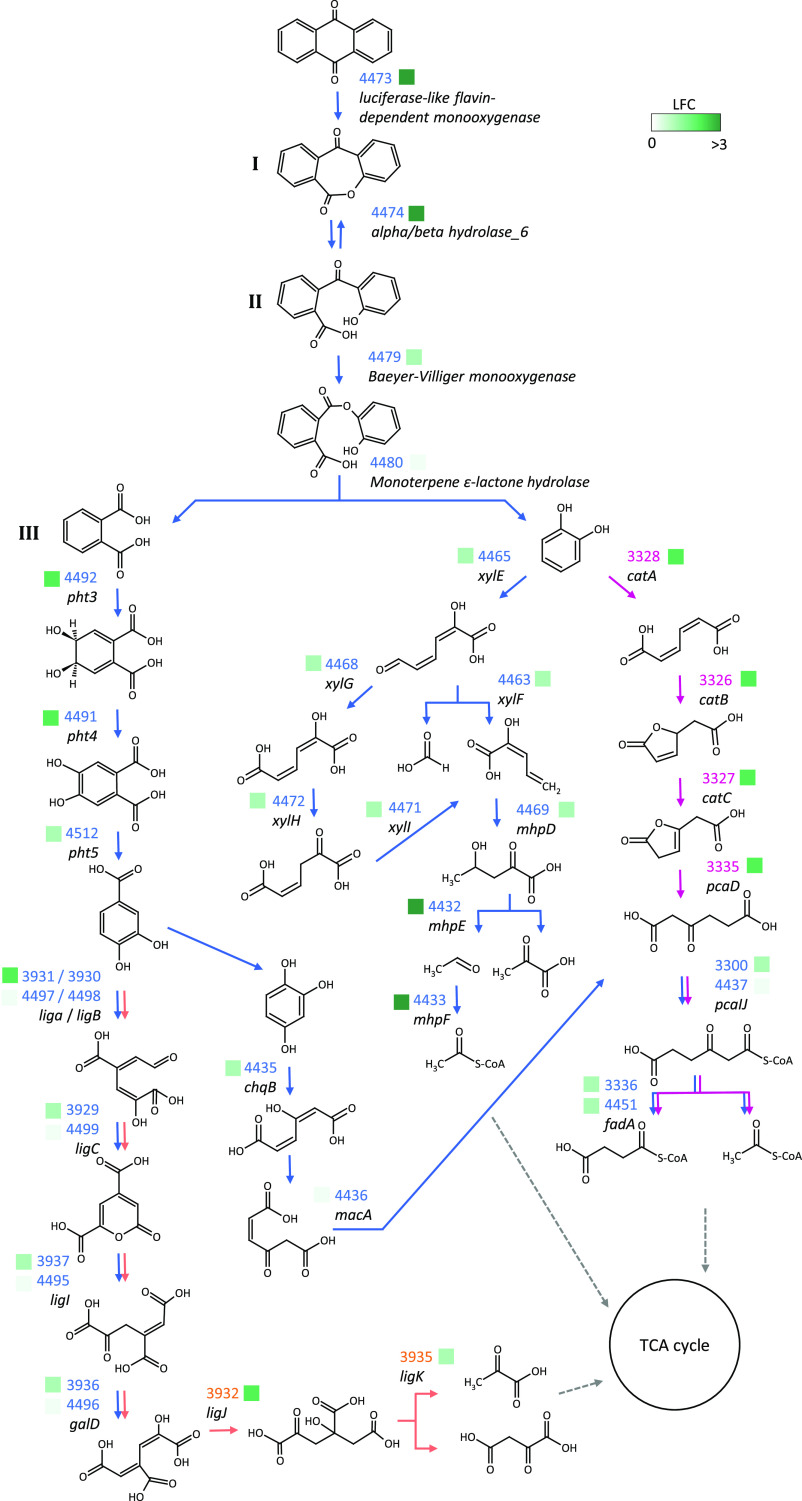
Schematic pathway proposed for the degradation of 9,10-anthraquinone by *Sphingobium* sp. strain AntQ-1. Roman numbers correspond to the detected metabolites. Pink arrows correspond to reactions catalyzed by enzymes encoded by genes located on the chromosome *chr1*; orange arrows correspond to genes on chromosome *chr2*; blue arrows correspond to genes on plasmid *pANTQ-1*. Gene IDs are indicated adjacent to the corresponding reactions following the same chromatic criterion. Squares in different shades of green represent the log_2_ fold change (LFC) of the corresponding genes in the RNA-Seq experiment when strain AntQ-1 grew on ANTQ.

The combination of metabolomic, genomic, and transcriptomic data provided a deep understanding of the ANTQ metabolic pathway in *Sphingobium* sp. AntQ-1 (Figure 2). The identification of dibenz[*b,e*]oxepin-6,11-dione, 2-(2-hydroxybenzoyl)-benzoic acid, and phthalic acid as major metabolites from anthraquinone degradation suggests that strain AntQ-1 metabolizes anthraquinone by cleavage of the central ring and further processing of the flanking aromatic rings to intermediates of the central metabolism. The attack would be initiated by a BV oxidation producing dibenz[*b,e*]oxepin-6,11-dione, a lactone that would be eventually hydrolyzed to 2-(2-hydroxybenzoyl)-benzoic acid. The overexpressed cluster of genes identified in the RNA-Seq analysis would orchestrate the initial attack of ANTQ, catalyzing the BV oxidation (sphantq_4473) and further hydrolysis of the resulting lactone (sphantq_4474).

Evidence of the formation of a lactone by BV oxidation has been documented for *Streptomyces aureofaciens* B-96, which transformed 1,8-dihydroxy-9,10-anthraquinone to give the corresponding lactone.^36^ Similar BV reactions have also been suggested from the identification of metabolites during the degradation of fluoranthene via acenaphthenone by *Alcaligenes denitrificans* WW1^37^ and fluorene via indanone by *Arthrobacter* sp. F101.^38,39^*Pseudomonas* sp. F274 and *P. mendocina* MC2 also produced a similar lactone during the degradation of 9-fluorenone; however, its formation was attributed to the cleavage of the central ring following an angular dioxygenation of one of the flanking aromatic rings of fluorene.^27,40^

The presence of a second cluster of BVMO–hydrolase coding genes and overexpression of both phthalate and catechol metabolic pathways suggests a second BV oxidation and hydrolysis of the detected 2-(2-hydroxybenzoyl)-benzoic acid leading to the formation of phthalate and catechol. In fact, catechol and an aminosulfo-derivative of phthalic acid were detected as biodegradation products from 1-aminoanthraquinone-2-sulfonic acid by a *Rhodococcus pyridinivorans* strain with the ability to transform a variety of anthraquinone derivatives.^41^

Essential genes for the biodegradation of anthraquinone are primarily located on the megaplasmid *pANTQ-1* (Figure 1), including genes related to the upper ANTQ degradation pathway, the phthalate metabolism, and the *meta*-cleavage pathway of catechol. However, these genes are not organized in coordinately regulated operons as they are scattered across the plasmid; moreover, some genes for the phthalate and the catechol *ortho*-cleavage pathways are located on the chromosomes. The observed genomic arrangement with catabolic pathways encoded in megaplasmids (>100 kbp)^42^ and with genes dispersed through sparse clusters in the genome^43^ is common in sphingomonads. Plasmid *pANTQ-1* also encompassed several genes coding for transposases and type IV secretion system components, raising the possibility for plasmid-mediated gene transfer. It has also been found that sphingomonads with the ability to degrade compounds that are converted to intermediates of the naphthalene and biphenyl pathways are able to change the position and orientation of certain conserved gene clusters in their genomes.^44^ These characteristics give sphingomonads the ability to adapt quickly and efficiently to novel compounds in the environment, conferring them exceptional degradative capabilities for a wide range of contaminants. *Sphingobium* sp. AntQ-1, however, utilizes a few aromatic substrates, which could be explained by the high specificity of its enzyme system.

The incapability of *Sphingobium* sp. AntQ-1 to degrade PAHs is explained for its lack of PAH ring-hydroxylating and ring-cleavage dioxygenases, having to initiate the attack of 9,10-anthraquinone at the central ring. To oxidatively break a carbon–carbon bond, BVMOs require one of the carbons to be part of a carbonyl group, but they are also known for their exquisite chemo-, regio-, and enantioselectivity.^45^ In fact, the action of BVMOs of strain AntQ-1 seem to be sterically hindered by larger structures such as the presence of a third aromatic ring in 7,12-benz(*a*)anthracenequinone. All this is consistent with its high specificity on polyaromatic substrates. If biodegradation of oxy-PAHs relies on highly specialized BVMOs, this could be the reason for their wide environmental distribution^46^ and accumulation during bioremediation of PAH-contaminated soils.

Of the tested monoaromatic substrates, AntQ-1 utilized those ready to enter the identified catechol and protocatechuic acid pathways except for phthalic acid. Although the strain has all the necessary enzymes for its further metabolism, the presence of the two carboxylic groups would hinder its diffusion through the lipophilic bacterial wall, needing specific transport mechanisms. This has also been observed for other PAH degrading strains with phthalic degradation pathways.^40^

### Environmental Relevance in PAH-Contaminated Soils

**Figure 3:**
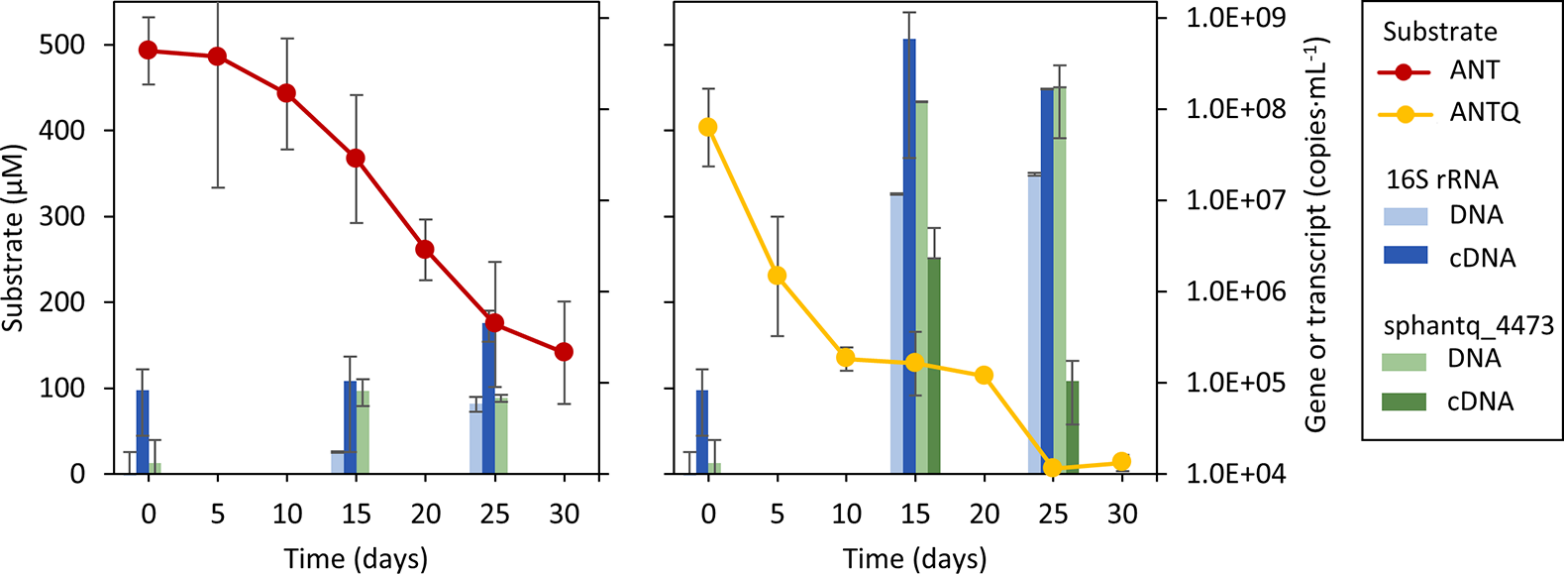
Biodegradation of anthracene (red) and 9,10-anthraquinone (yellow) in the sand-in-liquid soil microcosms and quantification of *Sphingobium* sp. AntQ-1 specific 16S rRNA (blue bars) and BVMO 4473 (green bars) gene copies (DNA, light color) and transcripts (cDNA, dark color) in the sand-in-liquid soil microcosms with either anthracene or anthraquinone at 0, 15, and 25 days of incubation.

The relevance of *Sphingobium* sp. AntQ-1 and its oxy-PAH degradation mechanism in the creosote-contaminated soil was assessed by specific qPCR quantification of its 16S rRNA and the BVMO-coding gene sphtantq_4473. DNA and cDNA samples from the sand-in-liquid soil microcosms spiked with either ANT or ANTQ at 0, 15, and 25 days of incubation were used to quantify the aforementioned genes and their transcripts (Figure 3). Significant increases in gene and transcript copy numbers of strain AntQ-1 16S rRNA and BVMO were detected after 15 days of incubation in the presence of ANTQ with respect to time 0. For BVMO gene and transcript copies, the levels increased by four and two orders of magnitude, respectively. It is noteworthy that their expression during the incubation with ANTQ correlated with the biodegradation rates as it decreased after 25 days when ANTQ was completely removed. In the ANT-spiked microcosms, no expression was detected for BVMO. However, a small increase of BVMO gene copies and *Sphingobium* sp. AntQ-1 transcripts was detected, indicating that a fraction of ANT would be transformed to ANTQ and channeled through the described ANTQ-degradation pathway.

Recent works have raised increasing awareness on the potential toxicity and persistence of transformation products of organic contaminants^6^ and reinforced the need to include such products in risk analysis. Specifically, during the remediation of PAH-contaminated soils, genotoxicity of the soil has been observed to eventually increase despite effective PAH removal, and this increased genotoxicity has been associated to oxy-PAH formation.^8,9^ Our work identifies the existence of highly specialized microbial communities in soils with the ability to degrade PAH transformation products and elucidates the bacterial mechanisms involved in the biodegradation of 9,10-anthraquinone, a model oxy-PAH frequently identified in PAH-contaminated environments. Quantification of BVMOs by qPCR could be an appropriate method to assess the potential of native microbial communities to cycle PAH-transformation products, therefore providing evidence of the capabilities of native microbial communities to mitigate the risk associated to oxy-PAH formation during remediation.

## Supporting Information Available

Additional information on experimental methods, including details on nucleic acid extractions from microcosms, PCR amplification, Sanger sequencing, and chemical analyses; community analysis of ANT and ANTQ microcosms; SEM images and growth of strain AntQ-1 on ANTQ; spectral data (GC–MS, HPLC-HRMS and NMR) of ANTQ metabolites; spectral data of anthrone metabolites (GC–MS and HPLC-HRMS); assembly graph of the *Sphingobium* sp. AntQ-1 genome; RNA-Seq and gene expression data; KEGG reconstruction of the vitamin B_12_ metabolic pathway (PDF)

## References

[ref1] Lundstedt S.; Bandowe B. A. M.; Wilcke W.; Boll E.; Christensen J. H.; Vila J.; Grifoll M.; Faure P.; Biache C.; Lorgeoux C.; Larsson M.; Frech Irgum K.; Ivarsson P.; Ricci M. First Intercomparison Study on the Analysis of Oxygenated Polycyclic Aromatic Hydrocarbons (Oxy-PAHs) and Nitrogen Heterocyclic Polycyclic Aromatic Compounds (N-PACs) in Contaminated Soil. TrAC, Trends Anal. Chem. 2014, 83–92. 10.1016/j.trac.2014.01.007.

[ref2] Lampi M. A.; Gurska J.; McDonald K. I. C.; Xie F.; Huang X. D.; Dixon D. G.; Greenberg B. M. Photoinduced Toxicity of Polycyclic Aromatic Hydrocarbons to Daphnia Magna: Ultraviolet-Mediated Effects and the Toxicity of Polycyclic Aromatic Hydrocarbon Photoproducts. Environ. Toxicol. Chem. 2006, 25, 1079–1087. 10.1897/05-276R.1. 16629147

[ref3] Wilcke W.; Kiesewetter M.; Musa Bandowe B. A. Microbial Formation and Degradation of Oxygen-Containing Polycyclic Aromatic Hydrocarbons (OPAHs) in Soil during Short-Term Incubation. Environ. Pollut. 2014, 184, 385–390. 10.1016/j.envpol.2013.09.020. 24100048

[ref4] Biache C.; Ouali S.; Cébron A.; Lorgeoux C.; Colombano S.; Faure P. Bioremediation of PAH-Contamined Soils: Consequences on Formation and Degradation of Polar-Polycyclic Aromatic Compounds and Microbial Community Abundance. J. Hazard. Mater. 2017, 329, 1–10. 10.1016/j.jhazmat.2017.01.026. 28119192

[ref5] Andersson J. T.; Achten C. Time to Say Goodbye to the 16 EPA PAHs? Toward an Up-to-Date Use of PACs for Environmental Purposes. Polycyclic Aromat. Compd. 2015, 35, 330–354. 10.1080/10406638.2014.991042. PMC4714241 26823645

[ref6] Titaley I. A.; Simonich S. L. M.; Larsson M. Recent Advances in the Study of the Remediation of Polycyclic Aromatic Compound (PAC)-Contaminated Soils: Transformation Products, Toxicity, and Bioavailability Analyses. Environ. Sci. Technol. Lett. 2020, 7, 873–882. 10.1021/acs.estlett.0c00677. 35634165 PMC9139952

[ref7] Gillespie I. M. M.; Philp J. C. Bioremediation, an Environmental Remediation Technology for the Bioeconomy. Trends Biotechnol. 2013, 31, 329–332. 10.1016/j.tibtech.2013.01.015. 23427900

[ref8] Chibwe L.; Geier M. C.; Nakamura J.; Tanguay R. L.; Aitken M. D.; Simonich S. L. M. Aerobic Bioremediation of PAH Contaminated Soil Results in Increased Genotoxicity and Developmental Toxicity. Environ. Sci. Technol. 2015, 49, 13889–13898. 10.1021/acs.est.5b00499. 26200254 PMC4666737

[ref9] Tian Z.; Gold A.; Nakamura J.; Zhang Z.; Vila J.; Singleton D. R.; Collins L. B.; Aitken M. D. Nontarget Analysis Reveals a Bacterial Metabolite of Pyrene Implicated in the Genotoxicity of Contaminated Soil after Bioremediation. Environ. Sci. Technol. 2017, 51, 7091–7100. 10.1021/acs.est.7b01172. 28510420 PMC6309544

[ref10] Lundstedt S.; White P. A.; Lemieux C. L.; Lynes K. D.; Lambert I. B.; Öberg L.; Haglund P.; Tysklind M. Sources, Fate, and Toxic Hazards of Oxygenated Polycyclic Aromatic Hydrocarbons (PAHs) at PAH-Contaminated Sites. Ambio 2007, 36, 475–485. 10.1579/0044-7447(2007)36[475:SFATHO]2.0.CO;2. 17985702

[ref11] Larsson M.; Lam M. M.; Van Hees P.; Giesy J. P.; Engwall M. Occurrence and Leachability of Polycyclic Aromatic Compounds in Contaminated Soils: Chemical and Bioanalytical Characterization. Sci. Total Environ. 2018, 622-623, 1476–1484. 10.1016/j.scitotenv.2017.12.015. 29890612

[ref12] Idowu O.; Semple K. T.; Ramadass K.; O’Connor W.; Hansbro P.; Thavamani P. Beyond the Obvious: Environmental Health Implications of Polar Polycyclic Aromatic Hydrocarbons. Environ. Int. 2019, 123, 543–557. 10.1016/j.envint.2018.12.051. 30622079

[ref13] Clergé A.; Le Goff J.; Lopez C.; Ledauphin J.; Delépée R. Oxy-PAHs: Occurrence in the Environment and Potential Genotoxic/Mutagenic Risk Assessment for Human Health. Crit. Rev. Toxicol. 2019, 49, 302–328. 10.1080/10408444.2019.1605333. 31512557

[ref14] Moody J. D.; Freeman J. P.; Doerge D. R.; Cerniglia C. E. Degradation of Phenanthrene and Anthracene by Cell Suspensions of Mycobacterium Sp. Strain PYR-1. Appl. Environ. Microbiol. 2001, 67, 1476–1483. 10.1128/AEM.67.4.1476-1483.2001. 11282593 PMC92757

[ref15] Moody J. D.; Freeman J. P.; Cerniglia C. E. Degradation of Benz[a]Anthracene by Mycobacterium Vanbaalenii Strain PYR-1. Biodegradation 2005, 16, 513–526. 10.1007/s10532-004-7217-1. 15865344

[ref16] IARC. Anthraquinone. In IARC Monographs on the Evaluation of Carcinogenic Risks to Humans - Vol. 101; International Agency for Research on Cancer: Lyon, 2013; pp. 41–70. PMC7681469 1683674

[ref17] Vila J.; López Z.; Sabaté J.; Minguillón C.; Solanas A. M.; Grifoll M. Identification of a Novel Metabolite in the Degradation of Pyrene by Mycobacterium Sp. Strain AP1: Actions of the Isolate on Two- and Three-Ring Polycyclic Aromatic Hydrocarbons. Appl. Environ. Microbiol. 2001, 67, 5497–5505. 10.1128/AEM.67.12.5497-5505.2001. 11722898 PMC93335

[ref18] Lundstedt S.; Haglund P.; Öberg L. Degradation and Formation of Polycyclic Aromatic Compounds during Bioslurry Treatment of an Aged Gasworks Soil. Environ. Toxicol. Chem. 2003, 22, 1413–1420. 10.1897/1551-5028(2003)22<1413:DAFOPA>2.0.CO;2. 12836964

[ref19] Hu J.; Adrion A. C.; Nakamura J.; Shea D.; Aitken M. D. Bioavailability of (Geno)Toxic Contaminants in Polycyclic Aromatic Hydrocarbon–Contaminated Soil Before and After Biological Treatment. Environ. Eng. Sci. 2014, 31, 176–182. 10.1089/ees.2013.0409. 24803838 PMC3993035

[ref20] Rodgers-Vieira E. A.; Zhang Z.; Adrion A. C.; Gold A.; Aitken M. D. Identification of Anthraquinone-Degrading Bacteria in Soil Contaminated with Polycyclic Aromatic Hydrocarbons. Appl. Environ. Microbiol. 2015, 81, 3775–3781. 10.1128/AEM.00033-15. 25819957 PMC4421061

[ref21] Hareland W. A.; Crawford R. L.; Chapman P. J.; Dagley S. Metabolic Function and Properties of 4-Hydroxyphenylacetic Acid 1-Hydroxylase from Pseudomonas Acidovorans. J. Bacteriol. 1975, 121, 272–285. 10.1128/jb.121.1.272-285.1975. 234937 PMC285641

[ref22] Tauler M.; Vila J.; Nieto J. M.; Grifoll M. Key High Molecular Weight PAH-Degrading Bacteria in a Soil Consortium Enriched Using a Sand-in-Liquid Microcosm System. Appl. Microbiol. Biotechnol. 2016, 100, 3321–3336. 10.1007/s00253-015-7195-8. 26637425

[ref23] Weisburg W. G.; Barns S. M.; Pelletier D. A.; Lane D. J. 16S Ribosomal DNA Amplification for Phylogenetic Study. J. Bacteriol. 1991, 173, 697–703. 10.1128/jb.173.2.697-703.1991. 1987160 PMC207061

[ref24] Daniels L.; Handson R.; Philips J. Chemical Analysis. In Methods for general and molecular bacteriology; Gerhardt A., Murray R., Wood W., Krieg N., Eds.; ASM Press: Washington, DC, 1994; pp. 512–554.

[ref25] Love M. I.; Huber W.; Anders S. Moderated Estimation of Fold Change and Dispersion for RNA-Seq Data with DESeq2. Genome Biol. 2014, 15, 550 10.1186/s13059-014-0550-8. 25516281 PMC4302049

[ref26] Schmittgen T. D.; Livak K. J. Analyzing Real-Time PCR Data by the Comparative CT Method. Nat. Protoc. 2008, 3, 1101–1108. 10.1038/nprot.2008.73. 18546601

[ref27] Casellas M.; Grifoll M.; Sabaté J.; Solanas A. M. Isolation and Characterization of a 9-Fluorenone-Degrading Bacterial Strain and Its Role in Synergistic Degradation of Fluorene by a Consortium. Can. J. Microbiol. 1998, 734 10.1139/w98-066.

[ref28] Cajthaml T.; Möder M.; Kačer P.; Šašek V.; Popp P. Study of Fungal Degradation Products of Polycyclic Aromatic Hydrocarbons Using Gas Chromatography with Ion Trap Mass Spectrometry Detection. J. Chromatogr. A 2002, 974, 213–222. 10.1016/S0021-9673(02)00904-4. 12458938

[ref29] Baborová P.; Möder M.; Baldrian P.; Cajthamlová K.; Cajthaml T. Purification of a New Manganese Peroxidase of the White-Rot Fungus Irpex Lacteus, and Degradation of Polycyclic Aromatic Hydrocarbons by the Enzyme. Res. Microbiol. 2006, 157, 248–253. 10.1016/j.resmic.2005.09.001. 16256312

[ref30] Lu H.; Zhou J.; Wang J.; Liu G.; Zhao L. Decolorization of 1-Aminoanthraquinone-2-Sulfonic Acid by Sphingomonas Xenophaga. World J. Microbiol. Biotechnol. 2008, 24, 1147–1152. 10.1007/s11274-007-9586-1.

[ref31] Tolmie C.; Smit M. S.; Opperman D. J. Native Roles of Baeyer-Villiger Monooxygenases in the Microbial Metabolism of Natural Compounds. Nat. Product Rep. 2019, 36, 326–353. 10.1039/c8np00054a. 30074603

[ref32] Iwaki H.; Grosse S.; Bergeron H.; Leisch H.; Morley K.; Hasegawa Y.; Lau P. C. K. Camphor Pathway Redux: Functional Recombinant Expression of 2,5-and 3,6-Diketocamphane Monooxygenases of Pseudomonas Putida ATCC 17453 with Their Cognate Flavin Reductase Catalyzing Baeyer-Villiger Reactions. Appl. Environ. Microbiol. 2013, 79, 3282–3293. 10.1128/AEM.03958-12. 23524667 PMC3685261

[ref33] Leisch H.; Morley K.; Lau P. C. K. Baeyer-Villiger Monooxygenases: More than Just Green Chemistry. Chem. Rev. 2011, 111, 4165–4222. 10.1021/cr1003437. 21542563

[ref34] Maeda A. H.; Nishi S.; Hatada Y.; Ohta Y.; Misaka K.; Kunihiro M.; Mori J. F.; Kanaly R. A. Chemical and Genomic Analyses of Polycyclic Aromatic Hydrocarbon Biodegradation in Sphingobium Barthaii KK22 Reveals Divergent Pathways in Soil Sphingomonads. Int. Biodeterior. Biodegrad. 2020, 151 10.1016/j.ibiod.2020.104993.

[ref35] Perruchon C.; Vasileiadis S.; Papadopoulou E. S.; Karpouzas D. G. Genome-Based Metabolic Reconstruction Unravels the Key Role of B12 in Methionine Auxotrophy of an Ortho-Phenylphenol-Degrading Sphingomonas Haloaromaticamans. Front. Microbiol. 2019, 10, 1–12. 10.3389/fmicb.2019.03009. 31998277 PMC6970198

[ref36] Cudlín J.; Steinerová N.; Sedmera P.; Vokoun J. Microbial Analogy of Baeyer-Villiger Reaction with an Anthraquinone Derivative. Collect. Czech. Chem. Commun. 1978, 43, 1808–1810. 10.1135/cccc19781808.

[ref37] Weissenfels W. D.; Beyer M.; Klein J.; Rehm H. J. Microbial Metabolism of Fluoranthene: Isolation and Identification of Ring Fission Products. Appl. Microbiol. Biotechnol. 1991, 34, 528–535. 10.1007/BF00180583.

[ref38] Grifoll M.; Casellas M.; Bayona J. M.; Solanas A. M. Isolation and Characterization of a Fluorene-Degrading Bacterium: Identification of Ring Oxidation and Ring Fission Products. Appl. Environ. Microbiol. 1992, 58, 2910–2917. 10.1128/aem.58.9.2910-2917.1992. 1444405 PMC183026

[ref39] Casellas M.; Grifoll M.; Bayona J. M.; Solanas A. M. New Metabolites in the Degradation of Fluorene by Arthrobacter Sp. Strain F101. Appl. Environ. Microbiol. 1997, 63, 819–826. 10.1128/aem.63.3.819-826.1997. 9055403 PMC168377

[ref40] Grifoll M.; Selifonov S. A.; Chapman P. J. Evidence for a Novel Pathway in the Degradation of Fluorene by Pseudomonas Sp. Strain F274. Appl. Environ. Microbiol. 1994, 60, 2438–2449. 10.1128/aem.60.7.2438-2449.1994. 8074523 PMC201668

[ref41] Lu H.; Wang X.; Zang M.; Zhou J.; Wang J.; Guo W. Degradation Pathways and Kinetics of Anthraquinone Compounds along with Nitrate Removal by a Newly Isolated Rhodococcus Pyridinivorans GF3 under Aerobic Conditions. Bioresour. Technol. 2019, 285 10.1016/j.biortech.2019.121336. 30999187

[ref42] Stolz A. Degradative Plasmids from Sphingomonads. FEMS Microbiol. Lett. 2014, 350, 9–19. 10.1111/1574-6968.12283. 24111699

[ref43] Pinyakong O.; Habe H.; Omori T. The Unique Aromatic Catabolic Genes in Sphingomonads Degrading Polycyclic Aromatic Hydrocarbons (PAHs). J. Gen. Appl. Microbiol. 2003, 1–19. 10.2323/jgam.49.1. 12682862

[ref44] Basta T.; Buerger S.; Stolz A. Structural and Replicative Diversity of Large Plasmids from Sphingomonads That Degrade Polycylic Aromatic Compounds and Xenobiotics. Microbiology 2005, 151, 2025–2037. 10.1099/mic.0.27965-0. 15942009

[ref45] Mascotti M. L.; Lapadula W. J.; Ayub M. J. The Origin and Evolution of Baeyer - Villiger Monooxygenases (BVMOs): An Ancestral Family of Flavin Monooxygenases. PLoS One 2015, 10, 1–16. 10.1371/journal.pone.0132689. PMC4498894 26161776

[ref46] Bandowe B. A. M.; Wilcke W. Analysis of Polycyclic Aromatic Hydrocarbons and Their Oxygen-Containing Derivatives and Metabolites in Soils. J. Environ. Qual. 2010, 39, 1349–1358. 10.2134/jeq2009.0298. 20830923

