# Supplementary material for: Multi-Omic Profiling of a Newly Isolated Oxy-PAH Degrading Specialist from PAH-Contaminated Soil Reveals Bacterial Mechanisms to Mitigate the Risk Posed by Polar Transformation Products
Source: Environ Sci Technol. 2022 Dec 14;57(1):139–49. doi: 10.1021/acs.est.2c05485 (PMC9836352; doi:10.1021/acs.est.2c05485)
Supplement: Supplementary file 1 — es2c05485_si_001.pdf [file es2c05485_si_001.pdf]

Multi-omic profiling of a newly isolated oxy-PAH degrading specialist from PAH-contaminated soil reveals bacterial mechanisms to mitigate the risk posed by polar transformation products

Sara N. Jiménez-Volkerink[1]; Joaquim Vila[1*]; Maria Jordán[1], Cristina Minguillón[2]; Hauke Smidt[3]; Magdalena Grifoll[1]

[1] Department of Genetics, Microbiology and Statistics, University of Barcelona, Av. Diagonal, 643, 08028 Barcelona, Spain

[2] Department of Nutrition, Food Science and Gastronomy, University of Barcelona, Avda. Prat de la Riba, 171, 08921 Sta. Coloma de Gramanet, Barcelona, Spain

[3] Laboratory of Microbiology, Wageningen University & Research, P.O. Box 8033, 6700, EH Wageningen, the Netherlands

*corresponding author: Phone: +34 934034626, email: qvila@ub.edu.

Supplementary material

Number of pages: 21

Number of Tables: 7

Number of Figures: 13

Table of contents

Experimental methods S3

Chemical analyses S3

Nucleic acid extraction from sand-in-liquid soil microcosms and retro-transcription S3

PCR amplification, purification and Sanger sequencing S4

References S4

Table S1. Experimental design of the sand-in-liquid microcosms experiment S5

Table S2. Concentration of PACs in the creosote-contaminated soil. S6

Table S3. List of designed primers used for qPCR analysis. S7

Table S4. GC-MS and HPLC-MS properties of ANTQ metabolites from *Sphingobium* sp. AntQ-1. .... S8

Table S5. ^{1}H and ^{13}C NMR chemical shifts and coupling constants for metabolite II in $CDCl_3$ S9

Table S6. GC-MS and HPLC-MS properties of anthrone metabolites from *Sphingobium* sp. AntQ-1 ... S10

Table S7. Up-regulated genes related to the biodegradation of ANTQ by *Sphingobium* sp. AntQ-1. .... S11

Figure S1. DGGE profiles from the ANT and ANTQ-spiked sand-in-liquid soil microcosms. S12

Figure S2. (a) Image of *Sphingobium* sp. AntQ-1 grown on ANTQ in B_{12}-YE-MM agar plate. (b) Scanning electron microscopy (SEM) images of *Sphingobium* sp. AntQ-1 cells S13

Figure S3. Utilization of ANTQ by *Sphingobium* sp. AntQ-1 .... S14

Figure S4. Aromatic region of the ^{1}H NMR spectrum of metabolite II. S14

Figure S5. Aromatic region of the gCOSY spectrum of metabolite II. S15

Figure S6. Aromatic region of the gHSQC spectrum of metabolite II. S15

Figure S7. Assembly graph of the *Sphingobium* sp. AntQ-1 genome. S16

Figure S8. Phylogenomic tree of *Sphingobium* sp. AntQ-1 and other *Sphingobium* strains S17

Figure S9. Growth of *Sphingobium* sp. AntQ-1 in B_{12}-MM either with acetate or ANTQ S17

Figure S10. Volcano plot displaying the distribution of differentially expressed genes between acetate and ANTQ grown cultures of *Sphingobium* sp. AntQ-1. .... S18

Figure S11. Phylogenetic tree of Baeyer-Villiger monooxygenases (BVMOs) S19

Figure S12. KEGG reconstruction of the porphyrin metabolic pathway, including the biosynthesis of vitamin B_{12}, in *Sphingobium* sp. AntQ-1. .... S20

Figure S13. Fold change of selected differentially expressed genes by RNA-Seq and RT-qPCR S21

EXPERIMENTAL METHODS

Chemical analyses

Culture extracts were analyzed by GC with a flame ionization detector (GC-FID) on a TRACE 2000 GC (Thermo Quest, Italy) as described elsewhere[1]. PAHs and ANTQ were quantified by using five-point standard calibration curves. Extraction efficiency from the sand-in-liquid micrososms was 88% for ANT and 84% for ANTQ. GC-MS analyses were performed on an Agilent Technologies 6890N gas chromatograph coupled to a 5975 inert mass spectrometer as described elsewhere[1]. Prior to GC-MS analysis, the neutral and acidic extracts were derivatized with diazomethane and, when available, metabolites were identified by comparison with authentic standards.

HPLC-ESI-HRMS analysis was conducted on a UHPLC Ultimate 3000 (Thermo Scientific) chromatograph coupled to a PDA Accela (Thermo Fisher Scientific) detector set at 254 nm and a Linear Trap Quadropole (LTQ) Orbitrap Velos (Thermo Scientific) mass spectrometer with electrospray ionization (ESI) and positive polarity (+). Separation was achieved using an Accucore™ C18 column (Thermo Scientific) (100 x 2.1 mm; 2.6 µm particle size) and a linear gradient of methanol (10 to 95% [vol/vol] in 20 min) in acidified water (0.1% CH_2O_2). Flow was 0.2 $mL\cdot min^{-1}$. Injection volume was 10 µL.

The NMR experiments were performed on a Varian VNMRS500 spectrometer operating at 499.63 MHz in ^1H. The ^1H spectra were recorded using a standard sequence with a 45° observe pulse, delay recover 1s, 48 transients under controlled temperature (25 °C). The 2D experiment ^1H-^1H gCOSY (^1H-^1H gradient enhanced COrrelation SpectroscopY experiment) was acquired with 16 scans, 128 increments and the spectral width used was 13.7 ppm in the two dimensions. The ^1H-^{13}C heterocorrelation experiment was performed using a standard sequence gHSQC (gradient enhanced Heteronuclear Single Quantum Correlation). This spectrum was acquired with spectral windows of 13.7 ppm, for the ^1H dimension, and 199.9 ppm, for the ^{13}C dimension. A 1s prescan delay and 24 scans, 128 increments were used. The spectra were processed with the MestReNova program Version 12.03. The ^{13}C chemical shifts were extracted from the gHSQC analysis (only ^{13}C attached to ^1H are visible). The ^1H chemical shifts and ^1H coupling constants were obtained through a multiplet analysis routine included in the MestReNova software.

Nucleic acid extraction from sand-in-liquid soil microcosms and retro-transcription

Total DNA and RNA from the sand-in-liquid microcosms were extracted from two separate 1 mL aliquots of sand and liquid of each triplicate microcosm. DNA was extracted with the DNeasy PowerSoil Kit (Qiagen, USA) and for RNA extraction the RNeasy PowerMicrobiome Kit (Qiagen, USA) was used. Prior to analysis, RNA samples were treated with DNase I RNase-free (Thermo Fisher, USA) in a total reaction of 10 µL including 8 µL of RNA extract, 1 µL DNase

(1U) and 1 µL buffer. 1 µL EDTA 50 mM was added to inactivate DNase when the reaction was completed. The absence of contaminant DNA was confirmed by PCR using universal 16S rRNA primers 27F and 1492R. cDNA was obtained by reverse transcription of RNA extracts using the High Capacity cDNA Reverse Transcription Kit (Applied Biosystems, USA) with random hexamers.

PCR amplification, purification and Sanger sequencing

16S rRNA gene fragments were amplified by PCR using pureTaq Ready-To-Go PCR bead tubes (GE Healthcare, United Kingdom), in a final volume of 25 µL containing 1 µL DNA extract and 25 pmol of each primer (Sigma-Aldrich, Germany). Amplification was performed with primers GC40-63F and 518R[2] for DGGE analysis or primers 27F and 1492R[3] for strain identification. DNA or cDNA extracted from the sand-in-liquid soil microcosms or genomic DNA of the strain *Sphingobium* sp. AntQ-1 were used as template. PCR amplification was done on an Eppendorf Mastercycler and was validated with agarose gel (TBE 1x; 1% agarose) stained with Nancy 520 (Sigma-Aldrich, Germany). Sequencing of 16S rRNA amplification products was done by Macrogen Europe after purification with ExoSAP-IT (Thermo Fisher Scientific, USA). The resulting DNA sequences were manually adjusted using BioEdit v.7.2.5. and analyzed using the *Classifier* and S*equence Match* tools of the Ribosomal Database Project II[4] and the Blastn tool of GenBank.

References

(1) López, Z.; Vila, J.; Ortega-Calvo, J. J.; Grifoll, M. Simultaneous Biodegradation of Creosote-Polycyclic Aromatic Hydrocarbons by a Pyrene-Degrading Mycobacterium. *Appl. Microbiol. Biotechnol.* **2008**, *78* (1), 165–172. https://doi.org/10.1007/s00253-007-1284-2.

(2) Weisburg, W. G.; Barns, S. M.; Pelletier, D. A.; Lane, D. J. 16S Ribosomal DNA Amplification for Phylogenetic Study. *J. Bacteriol.* **1991**, *173* (2), 697–703. https://doi.org/10.1128/jb.173.2.697-703.1991.

(3) Maidak, B. L.; Cole, J. R.; Lilburn, T. G.; Parker, C. T.; Saxman, P. R.; Farris, R. J.; Garrity, G. M.; Olsen, G. J.; Schmidt, T. M.; Tiedje, J. M. The RDP-II (Ribosomal Database Project). *Nucleic Acids Res.* **2001**, *29* (1), 173–174. https://doi.org/10.1093/nar/29.1.173.

(4) Muyzer, G.; De Waal, E. C.; Uitterlinden, A. G. Profiling of Complex Microbial Populations by Denaturing Gradient Gel Electrophoresis Analysis of Polymerase Chain Reaction-Amplified Genes Coding for 16S RRNA. *Appl. Environ. Microbiol.* **1993**, *59* (3), 695–700. https://doi.org/10.1128/aem.59.3.695-700.1993.

Table S1. Experimental design of the sand-in-liquid microcosms experiment.

Compound	Condition	Inoculum[a]	Substrate concentration (µM)[b]	T0		T5		T10		T15		T20		T25		T30	
				C.A.[c]	M.C.[d]	C.A.	M.C.	C.A.	M.C.	C.A.	M.C.	C.A.	M.C.	C.A.	M.C.	C.A.	M.C.
**ANT**	**Culture**	2 ml	560	3	3	3	3	3	3	3	3	3	3	3	3	3	3
	**Control**	-	560	-	-	-	-	-	-	3	-	-	-	-	-	3	-
**ANTQ**	**Culture**	2 ml	480	3	3	3	3	3	3	3	3	3	3	3	3	3	3
	**Control**	-	480	-	-	-	-	-	-	3	-	-	-	-	-	3	-

[a]Sand-in-liquid microcosms were inoculated with 2 ml of a soil suspension, as described in the methods section.
bSubstrates were spiked at a final concentration of 0.1gL^{-1}, corresponding to the specified µM concentrations.
[c]C.A. Microcosms for chemical analysis. Number of replicates.
[d]M.C. Microcosms for microbial community analysis. Number of replicates.

Table S2. Concentration (mg·kg of dry soil^{-1}) of polycyclic aromatic compounds in the creosote-contaminated soil.

PAHs			
Naphthalene	563.4	±	12.5
Acenaphthylene	119.3	±	6.8
Acenaphthene	2276.9	±	4.5
Fluorene	2744.3	±	94.7
Phenanthrene	8865.4	±	65.5
Anthracene	3747.5	±	396.5
Fluoranthene	4288.8	±	32.9
Pyrene	2294.2	±	11.3
Benzo[*a*]anthracene	694.6	±	67.1
Chrysene	705.4	±	62.8
Benzo[*b*]fluoranthene	219.9	±	25.2
Benzo[*k*]fluoranthene	207.0	±	20.2
Benzo[*a*]pyrene	158.1	±	16.6
Benzo[*e*]pyrene	144.7	±	17.5
Indeno[*cd*]pyrene	46.2	±	6.1
Dibenzo[*ah*]anthracene	13.1	±	2.0
Benzo[*ghi*]perylene	84.2	±	8.1
∑17 PAHs	27173.0	±	600.0
LMW	18316.7	±	574.2
HMW	8856.2	±	167.7
N-PACs			
Methylquinoline	33.5	±	2.6
Dimethylquinoline	44.4	±	8.1
Benzo[*h*]quinoline	32.8	±	1.5
Acridine	339.7	±	30.5
Methylacridine	107.3	±	11.8
Carbazole	1839.3	±	414.0
Methylcarbazole	169.2	±	7.9
∑7 N-PACS	2568.0	±	366.0
Oxy-PAHs			
9-Fluorenone	40.9	±	1.1
Anthracene-9,10-dione	122.2	±	1.3
4H-Cyclopenta[*def*]phenanthrenone	17.7	±	1.3
2-Methylanthracene-9,10-one	20.0	±	2.0
Benz[*a*]fluorenone	22.7	±	0.7
Benz[*a*]anthracene-7,12-dione	6.6	±	0.3
Naphthacene-5,12-dione	43.8	±	2.7
∑7 Oxy-PAHs	273.0	±	9.0

* 5 g soil samples were combined with an equal amount of Na_2SO_4 and extracted in a Soxhlet apparatus (dichloromethane:acetone, 2:1) for 6h. The resulting organic extracts were concentrated to 5 ml and 1 ml aliquots were dried, transferred to 1 ml of hexane:dichlrorométhane (80:20 v/v), and loaded onto silicagel SPE columns for further fractionation. Two fractions (F1 and F2) were eluted in 2.5 g of silicagel activated at 125ºC for 24 h and deactivated with 5% water were packed into 20 mm (i.d.) glass SPE columns (BakerBond®, Deventer, The Netherlands). F1, eluted with 13 ml of hexane:dichloromethane (80:20, v/v) followed by 2 ml of dichloromethane, contained the PAHs; while F2, eluted with 1 ml of dichloromethane followed by 6 ml of methanol, contained the oxy-PAHs and N-PACs. Fractions were concentrated to 1 ml in dichloromethane and analyzed by GC as described in the Experimental Methods section. The quantified oxy-PAHs and N-PACs corresponded to those for which there were available reference standards according to Lundstedt et al. (2014) (http://dx.doi.org/10.1016/j.trac.2014.01.007). The quantified compounds accounted for 70% the total area of the chromatograms.

Table S3. List of designed primers used for qPCR analysis.

Target	Primer name	Sequence (5'>3')	Amplicon size (bp)	Annealing temp. (ºC)	qPCR standard curve Slope	qPCR standard curve R^2	qPCR efficiency (%)
16S rRNA *Sphingobium* sp. AntQ-1	sphantq-F	GGCCCGCTGGACAAGTATT	281	55	-2.929	0.998	119.507
	sphantq-R	GGAAATCGCGATGAGGATGTCA					
sphantq_4473	4473-F	AGTCCCGCACGAATTGTCAT	147	55	-3.467	0.994	94.286
Baeyer-Villiger monooxygenase	4473-R	ATGGGCACATCGAAAAAGCG					
sphantq_4474	4474-F	ATGAAGCCAACCCTGCACTT	102	55	-3.479	0.967	93.845
alpha/beta hydrolase	4474-R	TCTGCGCTGCCTTATACTCC					
sphantq_4479	4479-F	GAAGCATCGGGCGTCAAAAG	96	55	-4.270	0.994	71.480
Baeyer-Villiger monooxygenase	4479-R	CGCGAAGAGAGTCATACCCG					
sphantq_4480	4480-F	CGCGTTGCCCTTTATTTCCA	174	55	-3.642	0.998	88.179
Monoterpene ε-lactone hydrolase	4480-R	ATATGCCGCGATGCTGTCTT					
sphantq_4492	4492-F	TCGTCGCGCCTTATACTGTC	110	55	-3.510	0.992	92.719
Phthalate 4,5-dioxygenase oxygenase subunit	4492-R	ATGCCACGCAACCCAATAGA					
sphantq_3328	3328-F	GGACTCGCACGGCTTCTATT	167	55	-3.944	0.996	79.287
Catechol 1,2-dioxygenase	3328-R	CCTTCATCAGCGTGTCGGAT					
sphantq_4465	4465-F	AACTGTACCGCAAACCTCCC	121	55	-3.204	0.980	105.145
Catechol 2,3-dioxygenase	4465-R	GCCACGACGAAATCGAACAC					

Table S4. GC-MS Rt and EI mass spectral properties and HPLC-MS Rt and detected mass accuracies of major compounds formed from 9,10-anthraquinone by *Sphingobium* sp. strain AntQ-1 in neutral (N) and acidic (A) extracts.

		GC-MS				HPLC-MS			
	Extract	Rt (min)	Abundance (%)	*m/z* of fragment ions (% relative intensity)	Identification	Rt (min)	$[M+H]^+$	Molecular formula	δ (mmu)
I	N	26.7	21.9	224 (M+, 100), 196 (70), 168 (79), 152 (5), 139 (77), 126 (2), 114 (8), 104 (10), 92 (12), 76 (39), 63 (23), 50 (23)	dibenz[*b,e*]oxepin-6,11-dione	n.d.			
II	N<br>A	26.2	42.9<br>89.0	256 (M+, 39), 224 (100), 196 (83), 168 (52), 163 (12), 152 (5), 139 (29), 121 (33), 115 (12), 105 (8), 92 (16), 77 (22), 65 (25), 50 (8)	2-(2-hydroxybenzoyl)-benzoic acid ME	15.3	243.0649<br>243.0651	$C_{14}H_{11}O_4$	-0.2540<br>-0.1117
III	A	18.1	11.0	194 (M+, 6), 163 (100), 149 (1), 133 (6), 120 (2), 104 (5), 92 (8), 77 (20), 64 (2), 50 (6)	phthalic acid diME	2.1	149.0234	$C_8H_5O_3$	0.3102

Table S5. ^{1}H and ^{13}C NMR chemical shifts (δ) and coupling constants (J) for metabolite II in $CDCl_3$ (500 MHz).

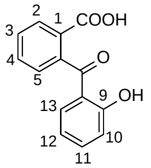

Assignment	δ (multiplicity; J)*
1-COOH	11.89
H-2	8.16 (dd; 7.8, 1.3 Hz)
H-3	7.61 (ddd; 7.8, 7.6, 1.3 Hz)
H-4	7.70 (ddd; 7.6, 7.6, 1.3 Hz)
H-5	7.38 (dd; 7.6, 1.3 Hz)
9-OH	2.01
H-10	7.05 (dd; 8.4, 1.1 Hz)
H-11	7.46 (ddd; 8.4, 7.2, 1.7 Hz)
H-12	6.76 (ddd; 8.1, 7.2, 1.1 Hz)
H-13	7.08 (dd, 8.1, 1.7 Hz)
C-2	131.1
C-3	129.8
C-4	133.2
C-5	127.4
C-10	118.2
C-11	136.4
C-12	118.9
C-13	132.6

*Chemical shifts (δ) are given in ppm. Multiplicity is indicated as dd (double doublet) or ddd (double double doublet) considering the appearance of signals in the spectrum. Coupling constants (J) are given in Hz.

Table S6. GC-MS Rt and EI mass spectral properties and HPLC-MS Rt and detected mass accuracies of major compounds formed from anthrone by *Sphingobium* sp. strain AntQ-1 in neutral (N) and acidic (A) extracts.

		GC-MS				HPLC-ESI(+)-HRMS			
	Extract	Rt (min)	Abundance (%)	*m/z* of fragment ions (% relative intensity)	Identification	Rt (min)	[M+H]+	Molecular formula	δ (mmu)
IV	N A	26.57	7.5 30.4	208 (M+, 100), 180 (100), 165 (4), 152 (81), 126 (10), 111 (2), 98 (4), 90 (4), 76 (33), 63 (8), 50 (14)	9,10-anthraquinone	18.5	209.0602	$C_{14}H_9O_2$	0.2134
II	A	26.74	22.3	256 (M+, 25), 223 (100), 196 (79), 168 (44), 163 (10), 152 (6), 139 (25), 121 (33), 115 (10), 104 (6), 92 (14), 77 (25), 65 (27), 50 (8)	2-(2-hydroxybenzoyl)-benzoic acid ME	15.4	243.0643	$C_{14}H_{11}O_4$	-0.8725
III	A	18.07	15.7	194 (M+, 6), 163 (100), 149 (1), 133 (6), 120 (2), 104 (5), 92 (8), 77 (20), 64 (2), 50 (6)	phtalic acid diME	2.15	149.0234	$C_8H_5O_3$	0.1054

Table S7. Up-regulated genes related to the biodegradation of 9,10-anthraquinone by *Sphingobium* sp. AntQ-1.

ID	Name	Description	Location	Length (bp)	log_2 fold change	Adjusted p-value	TPM
Upper ANTq pathway							
4473		Luciferase-like flavin-dependent monooxygenase	*pANTQ-1*	1041	4.05	2.7E-157	21461
4474		alpha/beta hydrolase_6	*pANTQ-1*	1125	3.49	3.0E-47	2570
4479		Baeyer-Villiger monooxygenase	*pANTQ-1*	1620	1.32	2.2E-13	80
4480		Monoterpene epsilon-lactone hydrolase	*pANTQ-1*	912	0.91	9.4E-04	7.2
Phthalate metabolism							
4492	*pht3*	Phthalate 4,5-dioxygenase oxygenase subunit	*pANTQ-1*	1272	2.36	2.1E-61	4782
4509	*pht2*	Phthalate 4,5-dioxygenase reductase subunit	*pANTQ-1*	963	1.59	2.3E-37	3965
4491	*pht4*	Phthalate 4,5-*cis*-dihydrodiol dehydrogenase	*pANTQ-1*	1197	2.30	8.7E-54	276
4512	*pht5*	4,5-dihydroxyphthalate decarboxylase	*pANTQ-1*	993	1.80	4.7E-34	2947
3931	*ligA*	Protocatechuate 4,5-dioxygenase alpha chain	*chr2*	456	2.08	8.2E-24	117
3930	*ligB*	Protocatechuate 4,5-dioxygenase beta chain	*chr2*	897	2.28	6.3E-34	105
4497	*ligA*	Protocatechuate 4,5-dioxygenase alpha chain	*pANTQ-1*	210	0.89	4.7E-02	37
4498	*ligB*	Protocatechuate 4,5-dioxygenase beta chain	*pANTQ-1*	843	0.86	6.9E-05	27
4435	*chqB*	Hydroxyquinol 1,2-dioxygenase	*pANTQ-1*	918	1.05	5.2E-06	47
4436	*macA*	Maleylacetate reductase	*pANTQ-1*	1065	0.72	3.4E-03	22
3929	*ligC*	4-carboxy-2-hydroxymuconate-6-semialdehyde dehydrogenase	*chr2*	942	1.80	1.0E-32	109
4499	*ligC*	4-carboxy-2-hydroxymuconate-6-semialdehyde dehydrogenase	*pANTQ-1*	942	0.18	4.7E-01	69
3937	*ligI*	2-pyrone-4,6-dicarboxylate hydrolase	*chr2*	873	1.58	1.2E-20	62
4495	*ligI*	2-pyrone-4,6-dicarboxylate hydrolase	*pANTQ-1*	888	0.96	1.8E-05	16
3936	*galD*	4-oxalomesaconate tautomerase	*chr2*	1062	1.81	5.3E-17	29
4496	*galD*	4-oxalomesaconate tautomerase	*pANTQ-1*	219	0.72	1.1E-01	7.9
3932	*ligJ*	4-oxalomesaconate hydratase	*chr2*	1026	2.51	1.9E-18	97
3935	*ligK*	4-carboxy-4-hydroxy-2-oxoadipate aldolase	*chr2*	675	1.97	1.6E-21	73
Catechol metabolism							
4465	*xylE*	Catechol 2,3-dioxygenase	*pANTQ-1*	924	1.38	1.3E-15	7055
4463	*xylF*	2-hydroxymuconate semialdehyde hydrolase	*pANTQ-1*	843	1.45	1.6E-15	301
4468	*xylG*	2-hydroxymuconate semialdehyde dehydrogenase	*pANTQ-1*	1458	1.41	4.9E-22	2252
4472	*xylH*	2-hydroxymuconate tautomerase	*pANTQ-1*	603	1.57	4.5E-14	1782
4471	*xylI*	4-oxalocrotonate decarboxylase	*pANTQ-1*	771	1.53	6.1E-26	1385
4469	*mhpD*	2-oxopent-4-enoate hydratase	*pANTQ-1*	807	1.50	8.6E-23	2830
4432	*mhpE*	4-hydroxy-2-oxovalerate aldolase	*pANTQ-1*	1047	4.49	2.9E-175	360
4433	*mhpF*	Acetaldehyde dehydrogenase	*pANTQ-1*	945	4.57	2.8E-139	533
3328	*catA*	Catechol 1,2-dioxygenase	*chr1*	927	2.21	8.7E-19	247
3326	*catB*	Muconate cycloisomerase	*chr1*	1158	2.43	4.7E-24	134
3327	*catC*	Muconolactone Delta-isomerase	*chr1*	291	2.08	1.6E-13	118
3335	*pcaD*	3-oxoadipate enol-lactonase	*chr1*	771	2.10	1.9E-13	36
3300	*pcaI*	3-oxoadipate CoA-transferase subunit alfa	*chr1*	687	-0.03	9.3E-01	5.3
4437	*pcaI*	3-oxoadipate CoA-transferase subunit alfa	*pANTQ-1*	585	1.05	2.6E-12	283
3336	*fadA*	acetyl-CoA acyltransferase	*chr1*	1206	1.90	1.3E-12	35
4451	*fadA*	acetyl-CoA acyltransferase	*pANTQ-1*	1257	1.05	2.1E-10	40

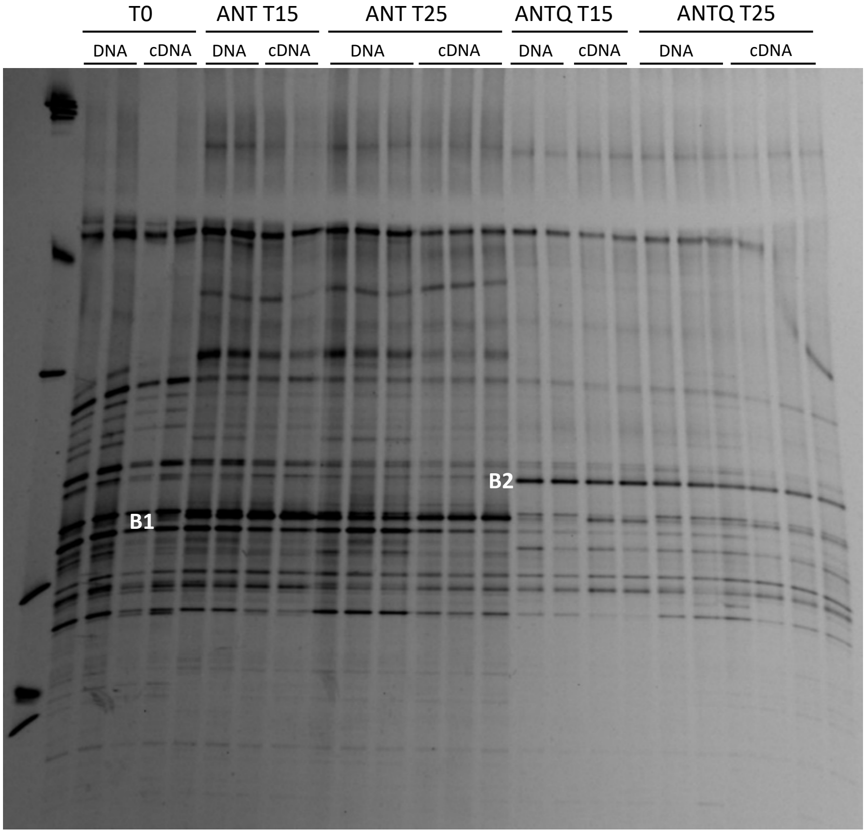

Band	Length (bp)	RDP Classification	Prob (%)	Blast closest match (Accession number)	Ident (%)
B1	321	*Sphingobium*	99	*Sphingobium fontiphilum* strain Chen16-4 (NR_109304)	99.7
B2	318	*Sphingobium*	100	*Sphingomonas bisphenolicum* strain AO1 (NR_153665)	100

Figure S1. DGGE profiles of the 16S rRNA gene fragments from DNA and cDNA samples from the ANT and ANTQ-spiked sand-in-liquid soil microcosms at 0, 15 and 25 days of incubation (T0, T15 and T25, respectively). Phylogenetic affiliation of the most relevant excised bands from the DGGE profiles of the ANT and ANTQ-spiked sand-in-liquid soil microcosms based on RDP database.

a)

b)

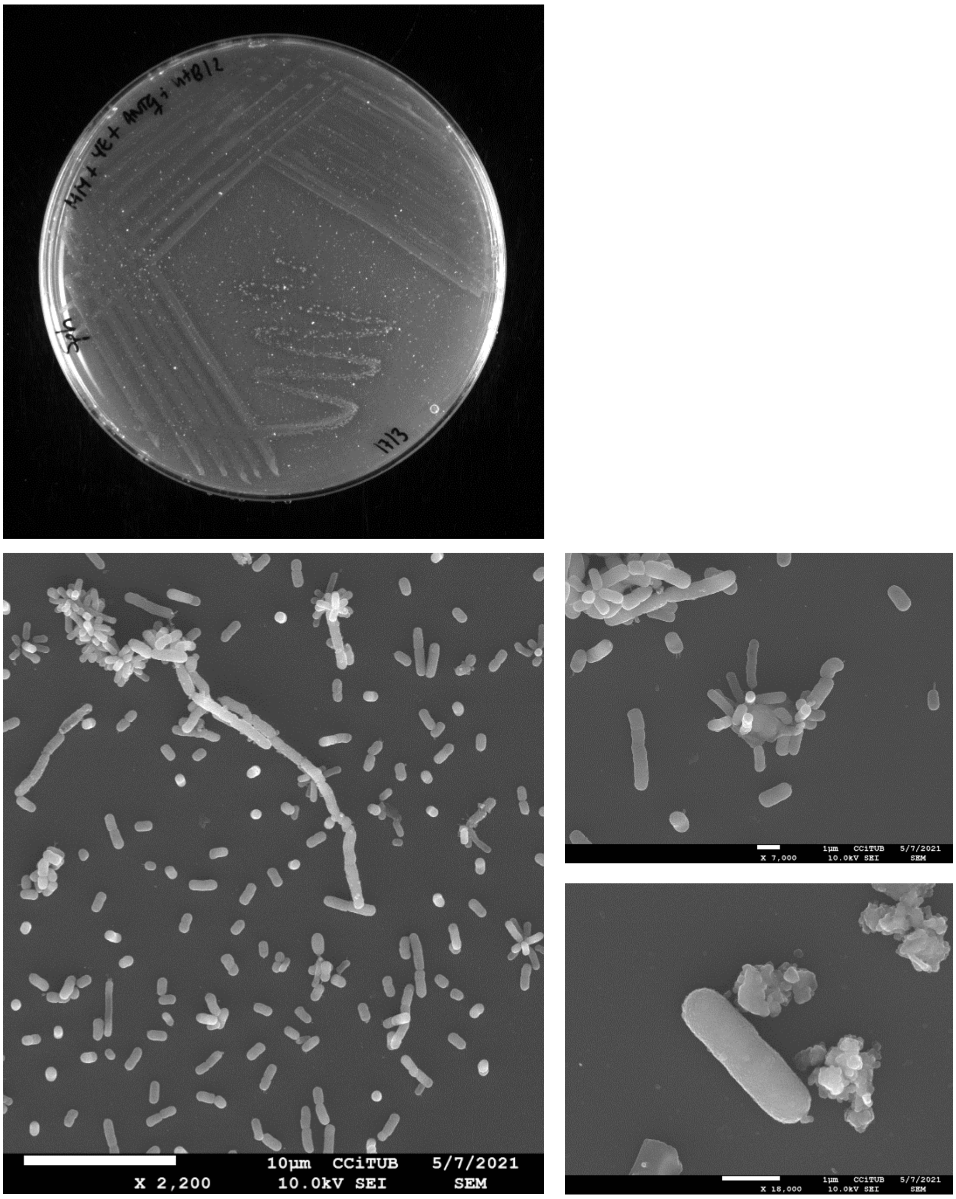

Figure S2. (a) Image of *Sphingobium* sp. AntQ-1 grown on a B_{12}-YE-MM agar plate containing ANTQ (0.1 g·L^{-1}) as main carbon source. Clearing areas around colonies indicating ANTQ degradation can be observed. (b) Scanning electron microscopy (SEM) images of *Sphingobium* sp. AntQ-1 cells grown with ANTQ as sole carbon source. Crystals of ANTQ are also visualized. SEM was performed at the Scientific and Technological Centres of the University of Barcelona (CCiTUB) on a JEOL JSM7001F microscope. Before observation, *Sphingobium* sp. strain AntQ-1 was grown in B_{12}-MM with ANTQ (0.1 g·L^{-1}) and the cells were fixated with 2% glutaraldehyde, subjected to critical point drying and coated with graphite.

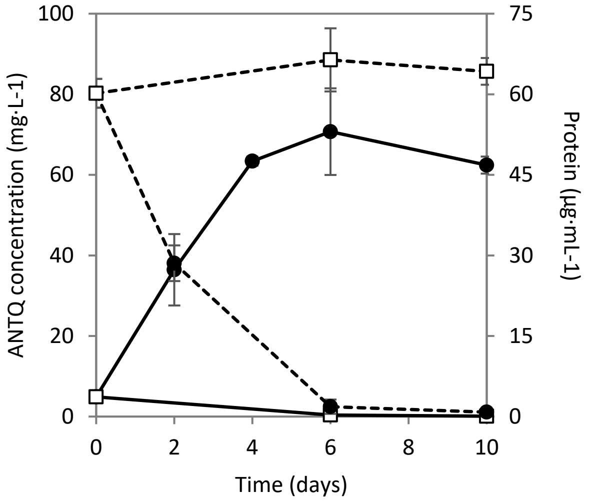

Figure S3. Utilization of 9,10-anthraquinone by *Sphingobium* sp. strain AntQ-1 in liquid mineral medium supplemented with vitamin B_{12} with anthraquinone as the sole source of carbon. Continuous lines correspond to protein concentration with (•) or without (□) ANTQ, and dashed lines correspond to ANTQ concentration in cultures with (•) or without inoculum (□).

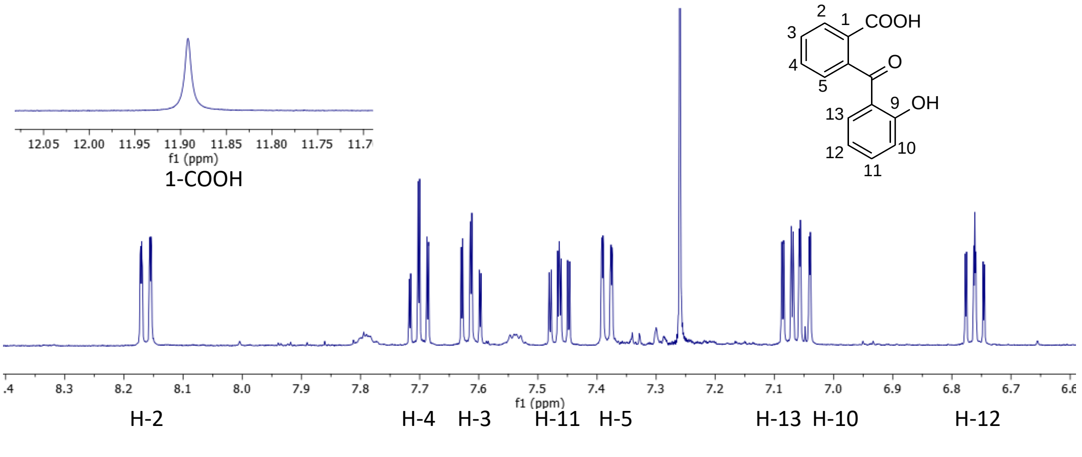

Figure S4. Aromatic region of the ^{1}H NMR spectrum of metabolite I.

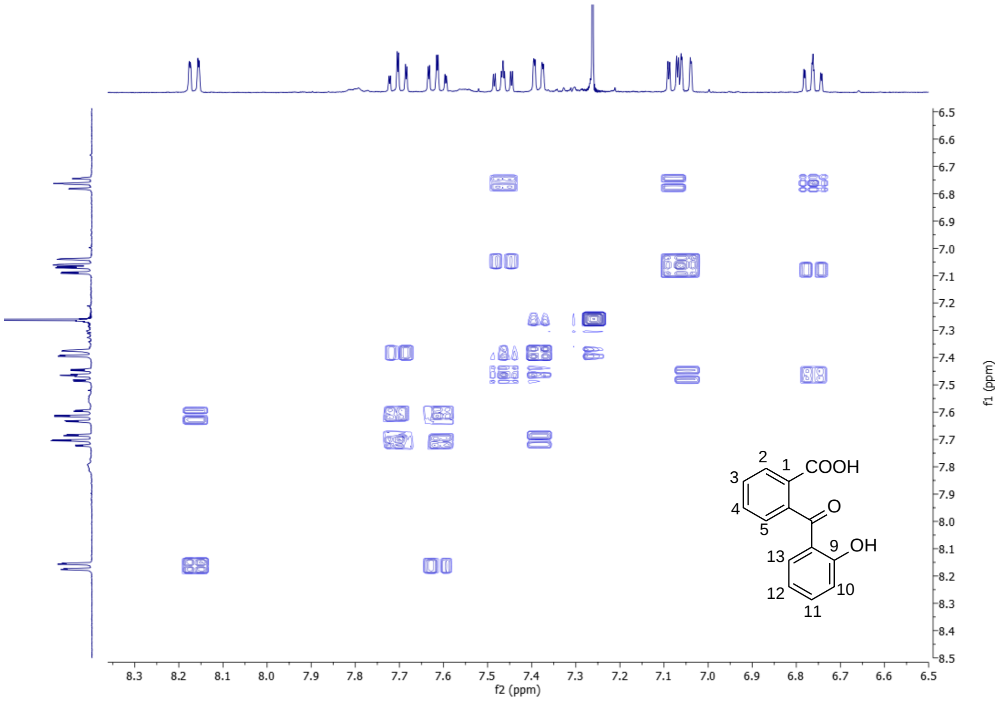

Figure S5. Aromatic region of the gCOSY spectrum of metabolite II.

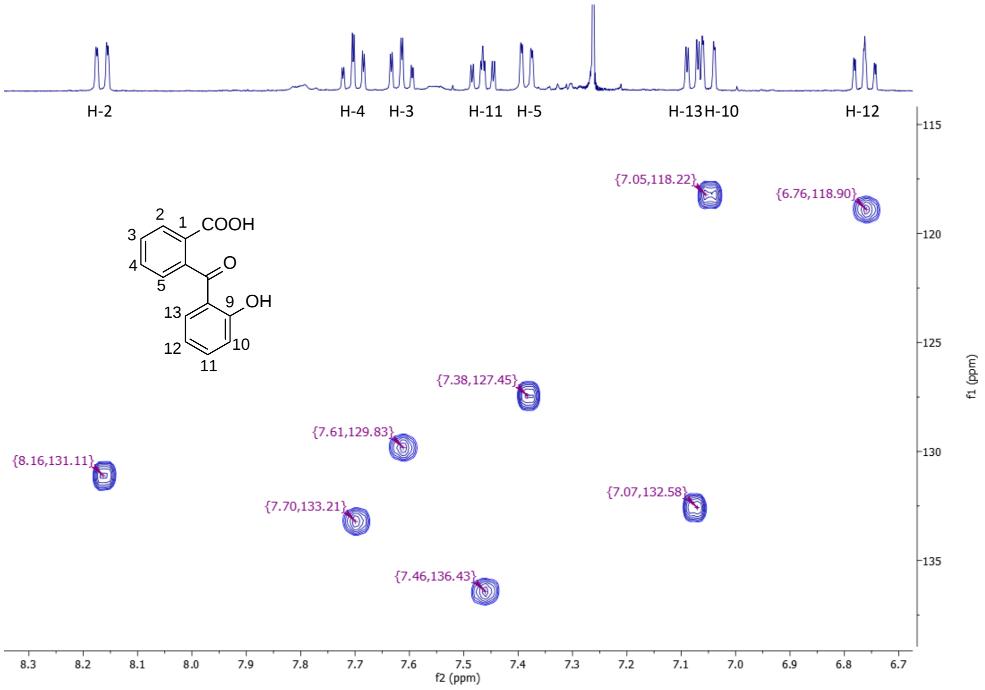

Figure S6. Aromatic region of the gHSQC spectrum of metabolite II.

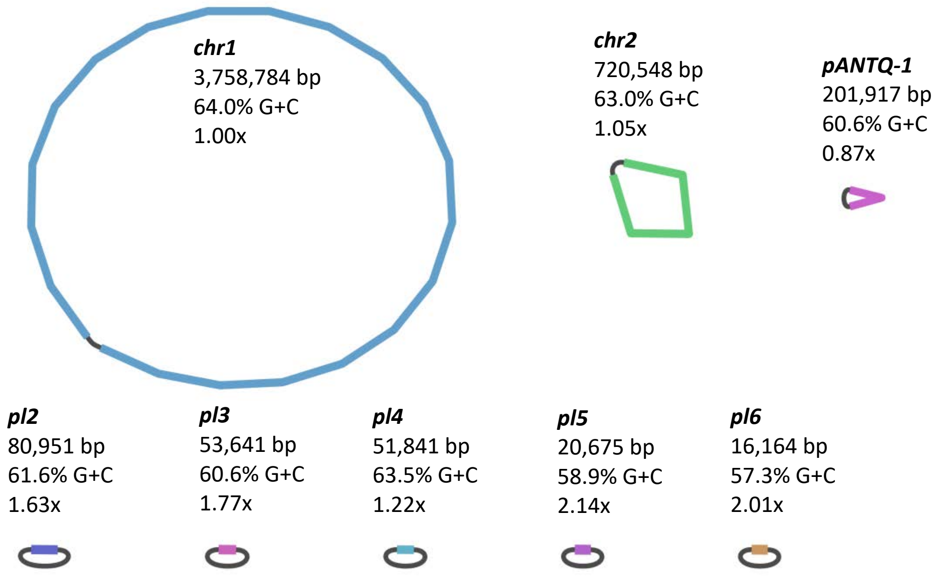

Figure S7. Assembly graph of the *Sphingobium* sp. AntQ-1 genome. Values given for each chromosome and plasmid are, from top to bottom, size in basepairs (bp), G+C percentage and coverage (x) in the sequence data.

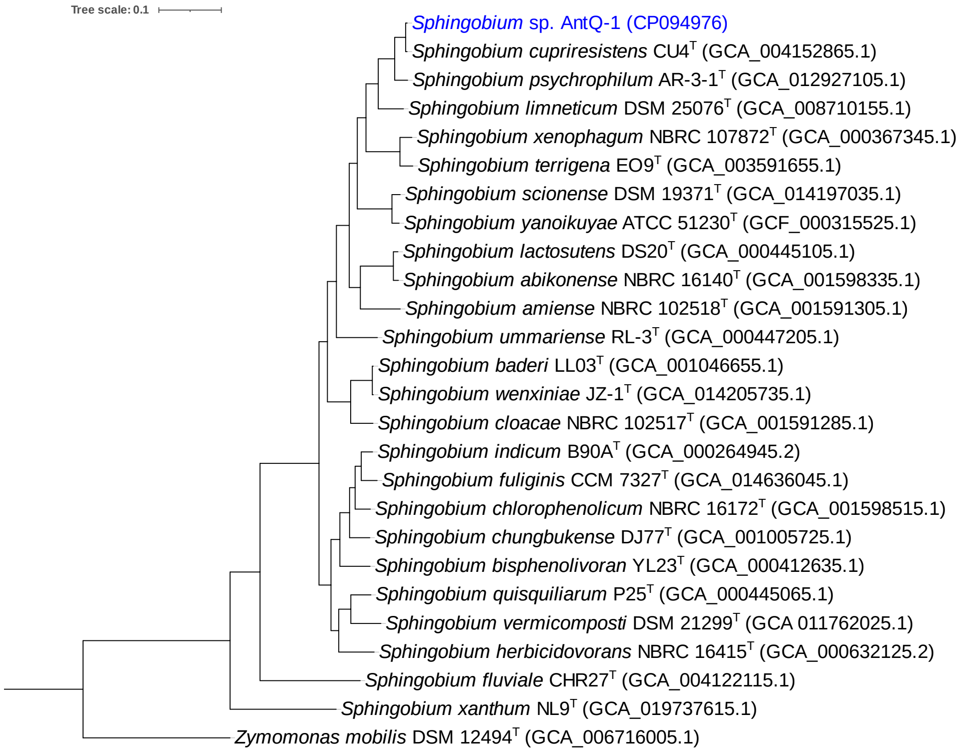

Figure S8. Phylogenomic tree of *Sphingobium* strains including *Sphingobium* sp. AntQ-1 (colored in blue). Phylogenetic tree was generated with GToTree v1.6.31, using the prepackaged single-copy gene-set unique to Alphaproteobacteria (117 target genes). Bootstrap values, calculated using 1,000 iterations, were of 100% for all branches. NCBI assembly accessions of the closest related genomes based on tetra-nucleotide signature correlation index performed with JSpeciesWS (only type strains were selected) were used as input files to build the de novo tree. *Zymomonas mobilis* (GCA_006716005.1) was used as an outgroup to root the tree. The final tree was generated and visualized on iTOL.

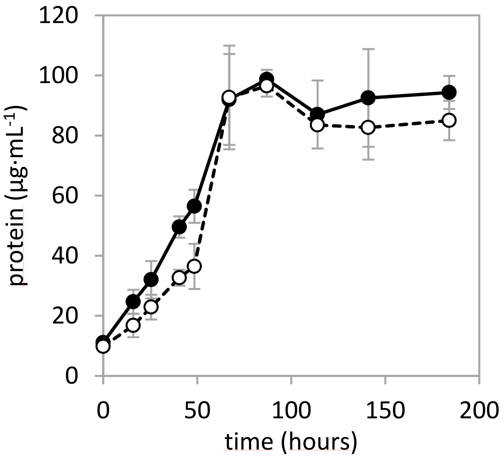

Figure S9. Growth of *Sphingobium* sp. strain AntQ-1 in liquid mineral medium supplemented with vitamin B_{12} either with acetate (○) or 9,10-anthraquinone (•) as the sole carbon source.

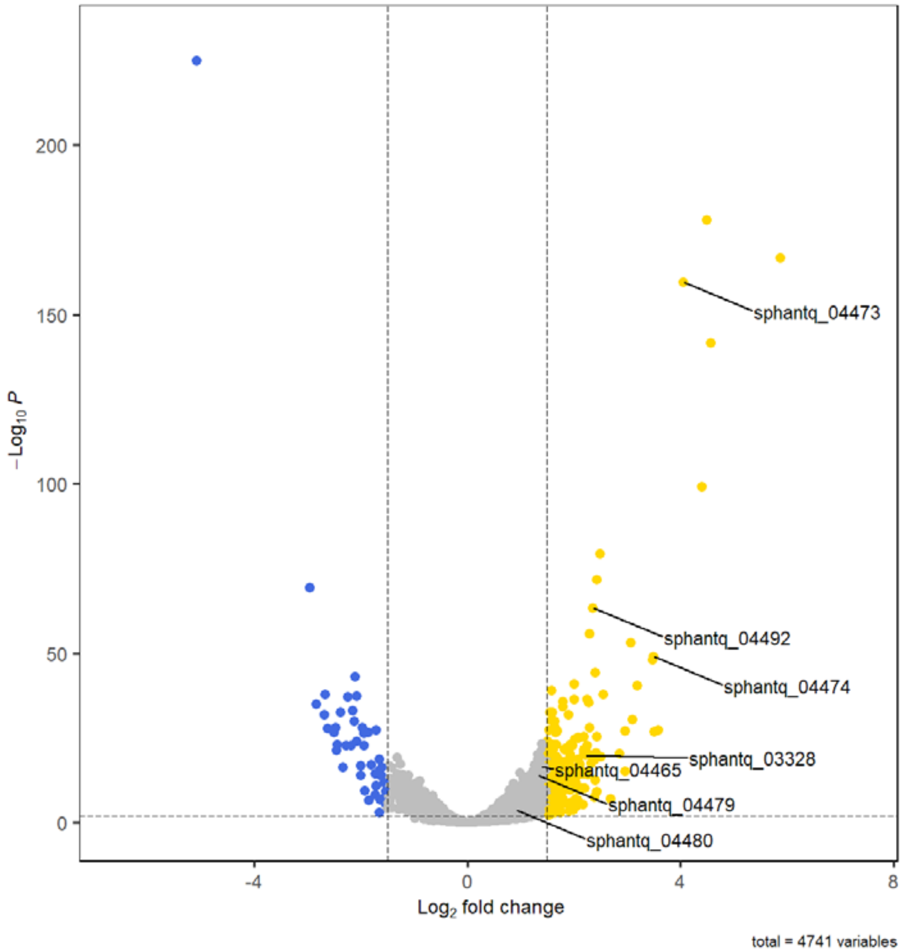

Figure S10. Volcano plot displaying the distribution of differentially expressed genes between acetate (blue) and anthraquinone (yellow) grown cultures of *Sphingobium* sp. strain AntQ-1. Positive log2 fold change indicates upregulation in anthraquinone cultures. The horizontal dashed line indicates adjusted p-value threshold = 0.01. The vertical dashed lines indicate log2 fold change threshold = 1.5.

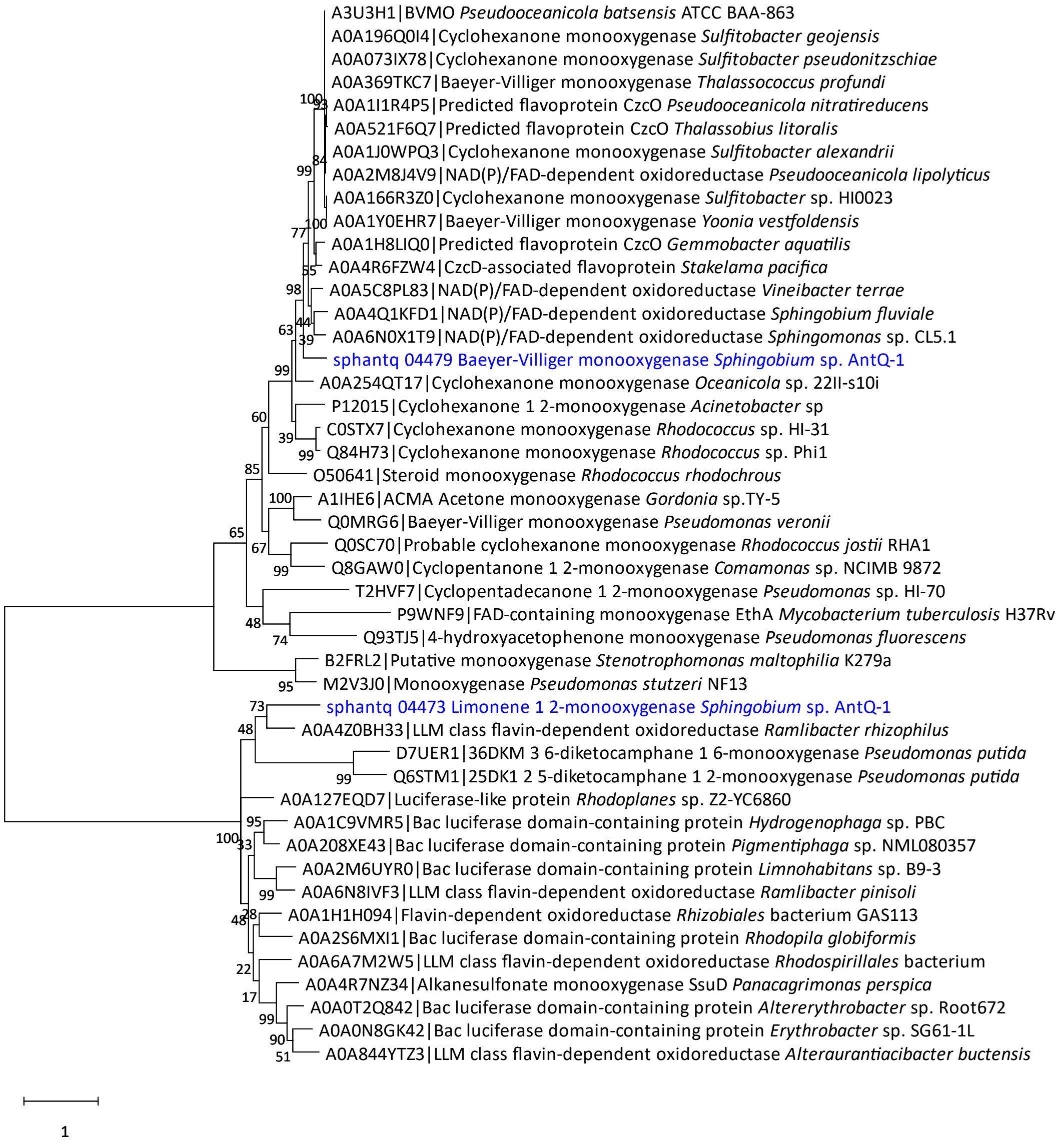

Figure S11. Phylogenetic tree of Baeyer-Villiger monooxygenases (BVMOs) using the Maximum Likelihood method and JTT matrix-based model. *Sphingobium* sp. AntQ-1 BVMOs sphantq_4473 and sphantq_4479 are in blue. Reference sequences corresponding to the closest monooxygenase protein sequences available in the UniProt database and well-characterized BVMOs are included. The tree is drawn to scale, with branch lengths measured in the number of substitutions per site. The tree was created with 1,000 bootstrap iterations. This analysis was performed in MEGA X and it involved 46 amino acid sequences. NCBI accession numbers are reported. Proteins sphantq_04473 and sphantq_04479 can be found in the genome of strain AntQ-1 (CP094976).

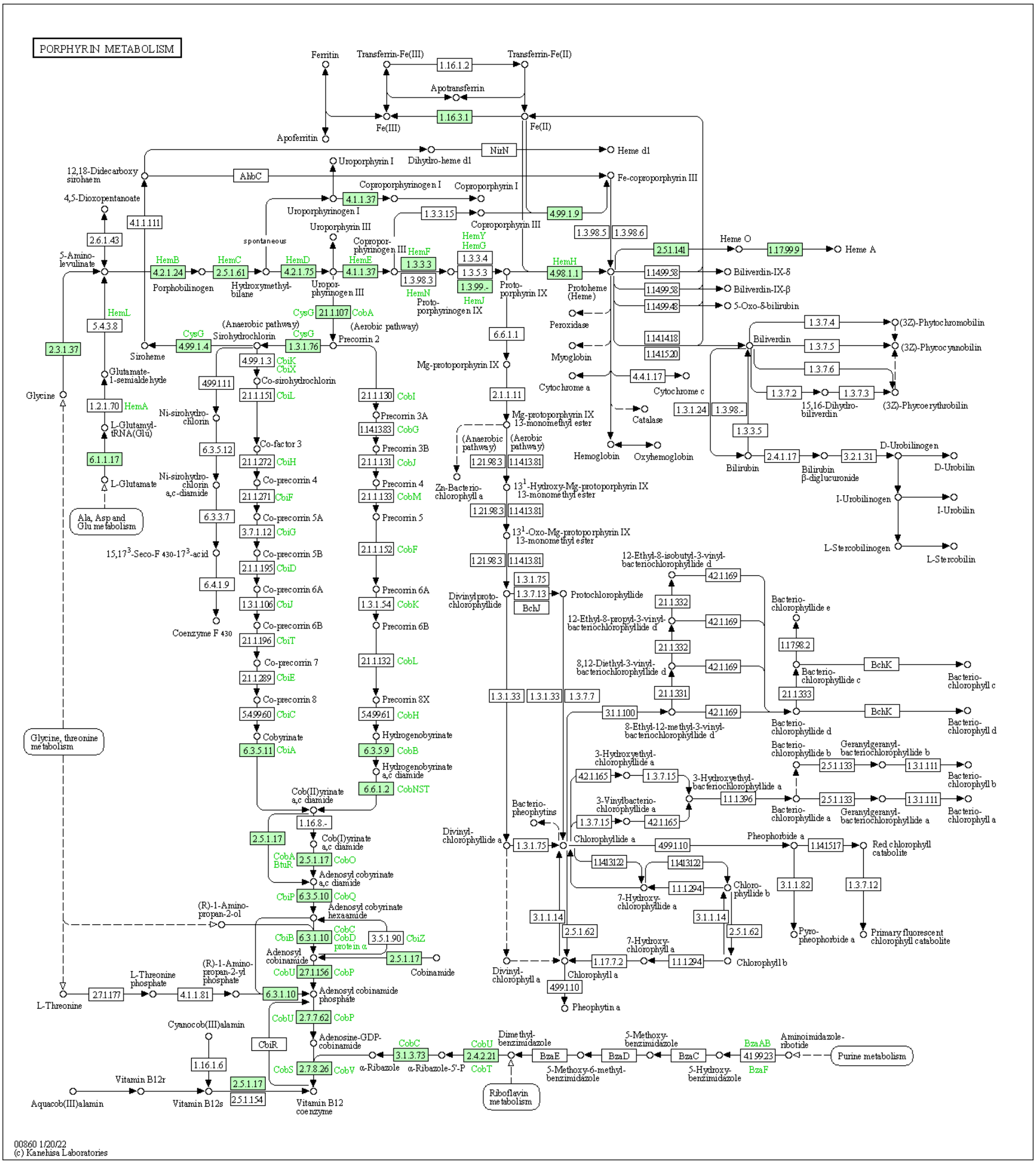

Figure S12. KEGG reconstruction of the porphyrin metabolic pathway, including the biosynthesis of vitamin B12, in *Sphingobium* sp. AntQ-1. Reconstruction was performed using the BlastKOALA tool with the amino acid sequences inferred from the genome annotation. Only the enzymes highlighted in green are encoded in the genome.

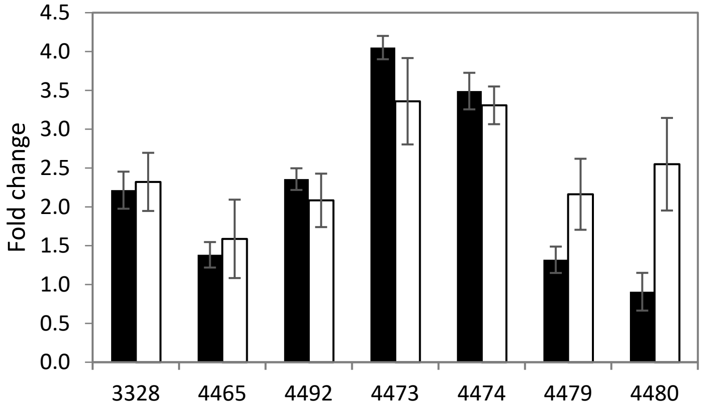

Figure S13. Fold change of selected differentially expressed genes by RNA-Seq, black bars, and RT-qPCR analysis, white bars. Values are means of three replicates and error bars represent standard deviation.
